# Preparation of new modified CuFe₂O₄ nanoparticles by benzalkonium chloride as enhancer of natural gas hydrate formation

**DOI:** 10.1038/s41598-026-44211-2

**Published:** 2026-04-22

**Authors:** A. M. Alsabagh, Abeer M. Shoaib, Mustafa Awad, S. A. Khalil, Mohamed S. Gad

**Affiliations:** 1 Process Development Dep., Egy. Pet. Res. Institute (EPRI), Nasr City, Cairo Egypt; 2 Applications Dep., (EPRI), Nasr City, Cairo Egypt; 3 Dep. of Petroleum. Refining and Petrochemical Engineering, Faculty of Petroleum and Mining Engineering, Suez University, Suez, 4351 Egypt

**Keywords:** Natural gas hydrate, Benzalkonium Chloride (Bzc), CuFe₂O₄, Phase equilibrium, Induction time, Rate of hydrate formation, NG storage, Chemistry, Environmental sciences, Materials science

## Abstract

Natural gas hydrate (NGH) based storage represents a safe and energy-efficient alternative to conventional natural gas transportation technologies; however, its widespread application remains constrained by slow hydrate formation kinetics and limited gas storage efficiency. Overcoming these limitations is essential for enabling hydrate-based gas storage as a practical industrial solution. In this context, the present study investigates the synergistic effect of benzalkonium chloride (Bzc) modified CuFe_2_O_4_ nanoparticles as an advanced kinetic promoter for NGH formation. Unmodified CuFe_2_O_4_ (NFCu), physically blended CuFe_2_O_4_/Bzc (NFCu-Phy.), and chemically modified CuFe_2_O_4_ Bzc (NFCu-Ch.) were systematically evaluated at 274.15 K and 6.5 MPa. The results demonstrate a substantial enhancement in hydrate formation kinetics and gas storage capacity with Bzc incorporation, particularly through chemical modification. NFCu exhibited an induction time of 12 min, while NFCu-Phy. and NFCu-Ch. reduced induction times to 6 and 5 min, respectively, at ultra-low Bzc dosages. NFCu-Ch. achieved a 58.33% reduction in induction time, indicating superior nucleation efficiency. Gas storage capacity increased markedly from 0.12 to 0.35 mol/mol water, corresponding to a 191.67% improvement. These enhancements are attributed to improved surface activity, favorable HOMOLUMO characteristics, and increased surface area and pore size. Overall, this study provides a low-dosage, high-efficiency strategy to overcome key kinetic and capacity barriers, supporting the practical advancement of NGH-based natural gas storage and transportation systems.

**Supplementary Information:**

The online version contains supplementary material available at 10.1038/s41598-026-44211-2.

## Introduction

Natural gas is essential for satisfying the world’s energy needs because it is widely available, highly efficient, and produces fewer environmental emissions than other fossil fuels. It serves as a cleaner energy source for electricity generation, industrial processes, and household use. One of the technologies for its storage and transportation is gas hydrate formation, which involves trapping natural gas molecules within water ice structures under specific pressure and temperature conditions. This method offers significant advantages, such as high energy density, safe storage, and the potential for long-distance transport in solid form. The advance of gas hydrate technologies enhances the efficiency and sustainability of natural gas utilization, especially in regions lacking pipeline infrastructure or facing challenges in conventional storage methods. Initial investigations into the part of nanoparticles in gas hydrate formation involved examining their impact on both nucleation and growth stages. To estimate what happened in two stages of gas hydrate formation process, Researchers employed high-pressure autoclaves and rocking cells to simulate the conditions under which hydrates form, often using methane or a natural gas mixture as the guest molecule^1^. Nanoparticles were used in these experiments included metal oxides (e.g., SiO₂, Al₂O₃, TiO₂), carbon-based materials (e.g., graphene, carbon nanotubes), and silica. These nanoparticles were chosen for their varying surface properties and ability to interact with the water phase. Nanoparticles have unique properties that significantly influence the gas hydrate formation process. Their impact on the formation and dissociation of hydrate can be attributed to several key functions and properties. The high surface area/volume ratio of nanoparticles significantly impacts hydrate formation. Research has shown that nanoparticles such as SiO₂ and TiO₂ offer abundant nucleation sites, which reduce the induction time and enhance the nucleation of methane hydrates^2^. Justifying that the large surface area of nano particles offers multiple points of contact for gas molecules and water to interact, facilitating the initial formation of the hydrate lattice. Furthermore, the high surface energy of nanoparticles also plays a vital role in prompting the nucleation process. researchers found that carbon nanotubes significantly accelerated the nucleation of methane hydrates by providing a favorable surface for the formation of hydrate crystals^3^. Moreover, nanoparticles can modify the thermodynamic stability of gas hydrates. Researches demonstrated that functionalized silica nanoparticles could alter the chemical potential of water, affecting the stability and the formation conditions of methane hydrates^4^. This modification can lead to changes in the conditions of temperature and pressure required for hydrate formation, which is significant in managing hydrate-related issues. The presence of nanoparticles can also affect the kinetics of hydrate formation and dissociation. The existence of nanoparticles can influence the rate of hydrate formation and dissociation by interacting with the growing hydrate structure. This interaction may impact heat and mass transfer processes, altering the kinetics of hydrate development and breakdown^5^. Hybrid nanoparticles with varying surface functionalities can either facilitate or hinder hydrate formation^6^. Functionalized nanoparticles with hydrophilic groups attracted water molecules and facilitated the formation of hydrate crystals, whereas hydrophobic modifications led to inhibition by preventing the proper alignment of water molecules^7^. The synergistic effects of nanoparticles and surfactants, were explored by that certain nanoparticle adsorbed gas molecules onto their surfaces, increasing the local gas concentration and promoting nucleation^8^. In addition, these materials can alter local environmental conditions, impacting hydrate formation. This change occurred due **to** nanoparticles could enhance the dissipation of heat generated during hydrate formation, stabilizing the growing crystals^9^. This ability could manage local heat and mass transfer conditions and help in controlling the growth of hydrate structures^10–12^. Nanoparticles at the gas-water interface influence the formation of hydrates by altering interfacial tension^13^. Various studies showed that well-dispersed nanoparticles provide a uniform distribution of nucleation sites, leading to more controlled and efficient hydrate formation^14^. Proper dispersion ensures that the nanoparticles can interact with the hydrate-forming system uniformly, enhancing or inhibiting formation as required^15,16^. Conversely, aggregation of nanoparticles can reduce their effective surface area and alter their impact on hydrate systems. Notably that how surfactants and other additives, including nanoparticles, could aggregate under certain conditions, potentially inhibiting hydrate formation by creating physical barriers around the growing hydrate crystals^1,17^. For more details that resulted in the survey, the effect of silica nanomaterials on the formation process of methane hydrate, concentrations ranging from 0.01 wt% to 0.5 wt% were used. The results indicated that at low concentrations (0.01 wt%), silica nanoparticles acted as promoters, enhancing the nucleation rate of methane hydrates^18^. However, In one experiment, researchers compared the effects of hydrophilic and hydrophobic silica nanomaterials on methane hydrate formation. They used concentrations of 0.1 wt% and observed that hydrophilic nanoparticles promoted hydrate nucleation, whereas hydrophobic ones acted as inhibitors^4,19^. Studies also showed that nanoparticles with high surface area and smaller pore sizes (e.g., mesoporous silica) could promote hydrate formation by providing more nucleation sites. For example, mesoporous silica nanoparticles with an average pore size of 2–4 nm were used in a study at a concentration of 0.1 wt%, resulting in a marked decrease in induction time compared to the control sample without nanoparticles^20^. Some nanoparticles, such as TiO₂ at a concentration of 0.05 wt%, were observed to lower the pressure required for hydrate formation by providing active sites for nucleation^21,22^. Furthermore, researchers have discovered that combining metal nanoparticles, such as silver, zinc oxide, and aluminum oxide, with a surfactant like SDS can significantly improve the promotion of natural gas hydrate formation. This blended approach has been shown to outperform the individual components, demonstrating the powerful synergistic effects that arise when surfactants and metal nanoparticles are used together in gas hydrate formation^23–25^. Magnetic nanoparticles (e.g., Fe₃O₄) have also been explored for their potential to be manipulated using external magnetic fields. In one study, magnetic nanoparticles at 0.1 wt% were used to control the nucleation process dynamically, offering a novel approach to managing hydrate formation in situ^26^. Using 500 ppm of SDS and 10 ppm of CuO, the suspension remained stable for five days. These nanoparticles notably reduced the induction time by 92.7% and increased methane hydrate gas storage capacity by 34%^27^. A combination of 0.035 wt% and 1.0 wt% of SDS and nano-CuO enabled the formation of methane hydrate to be completed in just 40 min, achieving a methane storage capacity of more than 170 v/v^9^. Scientists have examined the use of non-metal nanoparticles to enhance gas clathrate formation. These nanoparticles include graphite, graphene, graphene oxide, carbon nanotubes, and activated carbon. For example, when carbon nanotubes are added to a solution of SDS at a concentration of 0.2–0.6 mg/mL, the time it takes for hydrates to form is significantly reduced, from over 250 min to just 125–150 min. However, when oxidized carbon nanotubes are used instead, the promotion effect is even more pronounced, with hydrate formation occurring in as little as 100 min, even at a significantly lower concentration ranging from 0.01 to 0.1 mg/mL^28–31^. With using another type of surfactant (cationic surfactant) with nanometal, researchers studied how different concentrations of copper nanoparticles and CTAB solutions affect the process of forming methane hydrates. The findings indicated that both copper-based nanofluids and CTAB solutions were able to shorten the onset time of hydrate formation, with CTAB demonstrating greater effectiveness. All the additives tested had a positive impact on the total amount of methane used up and the hydrate growth rate^8,25,32,33^. Moreover, Researchers assessed the effects of Fe_3_O_4_ nanofluids with different concentrations of nanoparticles and additives (SDS and CTAB) on carbon dioxide hydrate formation^34^. The main goal of the present study is to investigate the enhancement role of cupper ferrite nano particles and its modified form by surfactant (Bzc) in the formation process of natural gas hydrates. The effectiveness of this modified formulation will be compared to copper ferrite alone, focusing on the phase equilibrium and the kinetics of natural gas hydrate (NGH) formation. The evaluation will also cover key aspects such as induction time during nucleation, gas consumption, hydrate formation rate, water-to-hydrate conversion efficiency, and total storage capacity.

## Experimental

### Material measurements and methods

#### Materials: used natural gas

The natural gas composition was analyzed using gas chromatography (GC) equipped with both a thermal conductivity detector (TCD) and a flame ionization detector (FID) to ensure precise measurement of its constituents. A representative sample was drawn into a sealed, gas-tight container and introduced into the GC via a gas injection loop. Helium served as the carrier gas, and specialized chromatographic columns were employed to effectively separate hydrocarbon and non-hydrocarbon components. Each compound was resolved based on its interaction with the column’s stationary phase and identified as distinct peaks in the chromatogram. Component identification and quantification were carried out by comparing retention times and peak areas with those of certified reference standards. The method accurately measured major constituents such as methane, ethane, propane, butanes, and pentanes, as well as trace components including nitrogen, carbon dioxide, and hydrogen sulfide. Results were reported in mole percent (mol%) and normalized to a total of 100% for consistency. Calibration procedures and quality assurance followed the ASTM D1945 and ISO 6974 guidelines, ensuring dependable measurements for evaluating energy content and overall gas quality^35^. The detailed composition of the natural gas sample is presented in Table 1.

**Table 1:** Natural Gas Composition.

Composition	C_1_	C_2_	C_3_	IC_4_	NC_4_	IC_5_	NC_5_	NC_6_	NC_7_	NC_8_	NC_9_	*N*_2_	CO_2_
**Mole %**	95.447	2.550	0.654	0.178	0.163	0.082	0.059	0.059	0.056	0.022	0.002	0.066	0.662

#### Used promoters

 Cationic surfactant (benzalkonium chloride, Bzc) and cupper ferrite (CuFe_2_O_4_) submitted from sigma Aldrich. Details regarding the source and purity of the surfactant and nanomaterials are provided in Table 2.

**Table 2:** Materials Used.

No.	`Material	Supplier	Purity, %
1	Natural gas mixture (methane)	Petrobel co. Aboumady fields, Egypt	95.447
2	Benzalkonium chloride (Bzc)	Aldrich sigma	99.5
3	Cu (NO_3_)_2_·3H_2_O	Aldrich sigma	99.5
4	Fe (NO_3_)_3_	Aldrich sigma	99.7

###  Preparation

**Fig. 1:**
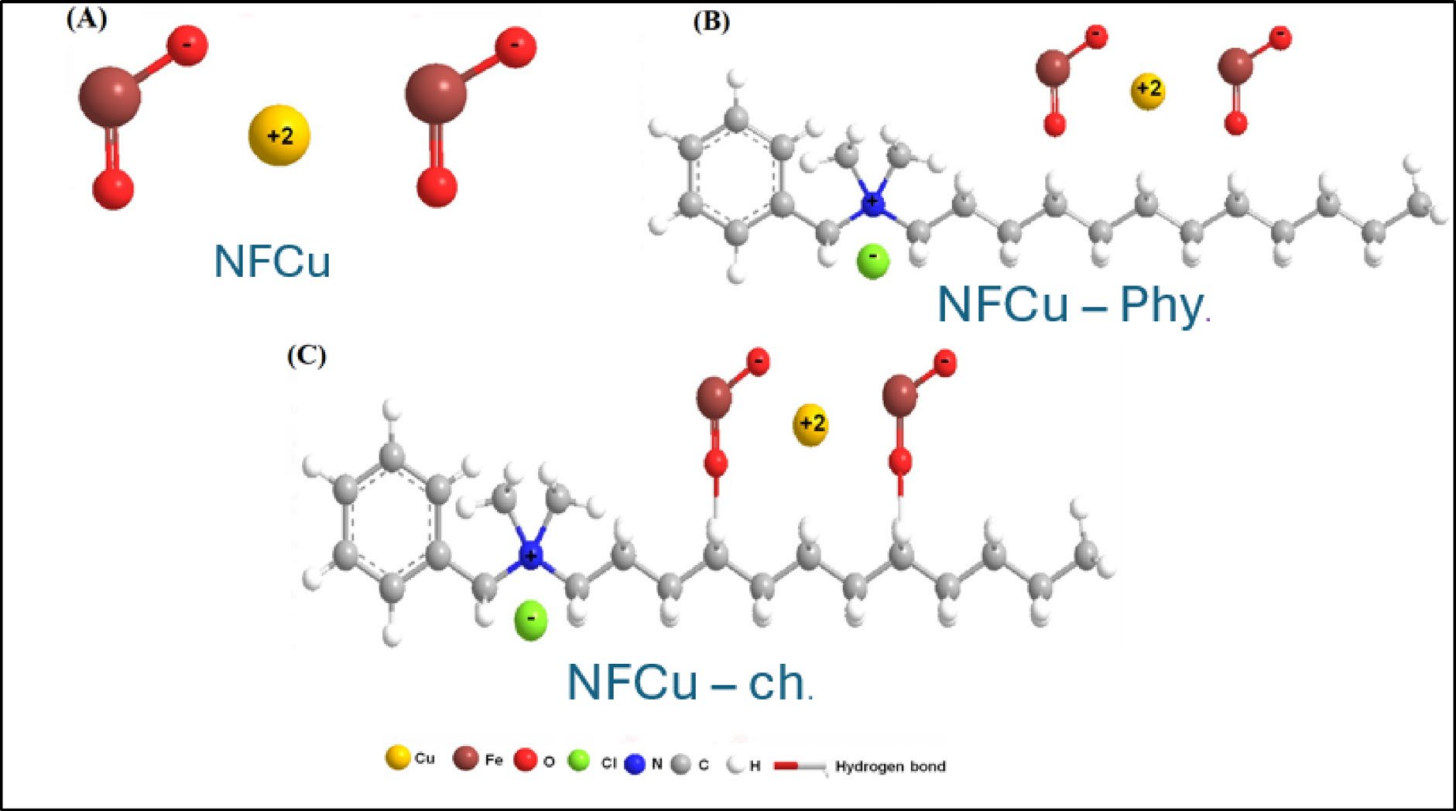
3D of Chemical Structure for **A** NFCu **B** NFCu- Phy. **C** NFCu- Ch.

The preparation process involved synthesizing three different nanofluid corrosion inhibitors. Figure 1 shows the 3D chemical structures of the prepared materials. (A) NFCu represents the base nanofluid copper structure. (B) NFCu-Phy refers to the nanofluid functionalized with a physical modifier. (C) NFCu-Ch illustrates the chemically modified nanofluid copper structure.

#### Preparation of CuFe_2_O_4_ nano particles

The CuFe_2_O_4_ was synthesized using co-perception method. Solution of copper (II) nitrate trihydrate 0.1 M (1.5 g) (Cu (NO_3_)_2_·3H_2_O) and a 0.2 M (5.1 g) solution of ferric nitrate (Fe (NO_3_)_3_) were mixed in a 1:2 molar ratio and the solution obtained was subjected to stirring for 30 min. Then, sodium hydroxide 1 M (NaOH) solution was added dropwise until the pH reaches between 10 and 12, to obtain a precipitate as a black-brown solid (yield 4.6 g,). The precipitate is left to settle for 1 h before being filtered and washed three times to remove any nitrate ions with 0.5 M NaOH and then with distilled water. Subsequent to washing, the precipitate was dried at 80 °C for 6 h to produce a dry powder of CuFe₂O₄, referred to as NFCu.

#### Physical blend of Bzc with CuFe_2_O_4_

Different concentrations of Bzc^35^ (ppm) blended with 0.02 wt% of NFCu. A physical blend was obtained generally named as NFCu- Phy.

#### Chemical modification of NF by Bzc

Copper ferrite (CuFe₂O₄) was synthesized via co-precipitation by mixing 0.1 M copper (II) nitrate and 0.2 M ferric nitrate under stirring for 30 min. At the same time an equal weight of Bzc (a cationic surfactant) was added to the precursor solution. Then, dropwise addition of 1 M sodium hydroxide solution was used to adjust the pH between 10 and 12, forming a complex black-brown CuFe₂O₄ - Bzc precipitate. The precipitate was allowed to settle, filtered, washed with 0.5 M NaOH and distilled water for three times, and finally dried at 80 °C for 6 h to get the fine powder NFCu- Ch. (yield, 33%).

### Chemical structure confirmation

#### FTIR spectroscopy

It was used to characterize the undertest compounds by using model [Fourier transform infrared spectroscopy (FT-IR)] at wavenumber range of 400–4000 cm⁻¹. The sample was prepared by method [Perkin Elmer spectrometer via the KBr pellet method (Spectrum One, USA)], and spectra were averaged over 32 scans at a resolution of 4 cm⁻¹.

#### X-ray diffraction (XRD)

The XRD measurements were conducted employing Cu Kα X-ray radiation (Germany, λ = 1.540 Å, PAN analytical X’PERT PRO) with a scan range of 5 < 2θ < 80.

#### Scan Electron Microscope (SEM)

The morphology and size of both compounds NFCu and NFCu-Ch. were characterized using a [Gemini SEM 500, Germany]. After being dispersed in ethanol, the nanoparticles were deposited onto a carbon-coated copper grid and dried under vacuum. Imaging was performed at a voltage of 15 kV and a working distance of 10 mm. The particles exhibited spherical morphology with an average size of [specific value, e.g., 25 ± 5 nm], as determined by analyzing SEM images using ImageJ software^36^.

#### The Brunauer–Emmett–Teller (BET) analysis

It is a crucial technique for determining the specific surface area and pore characteristics of the nanomaterials, which directly influence their performance in applications such as catalysis, sensing, adsorption, and energy storage.

### Apparatus setup

**Fig. 2:**
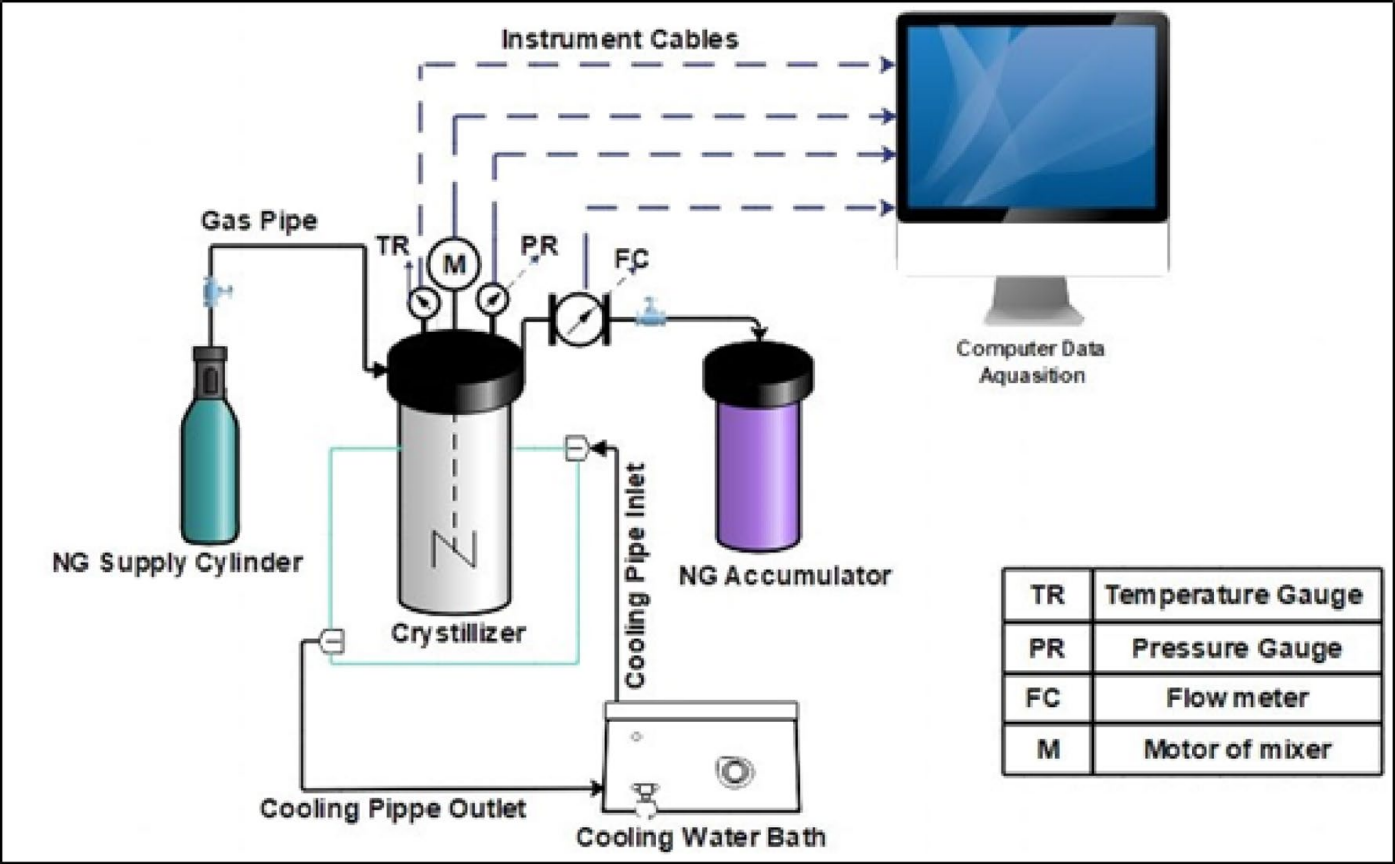
Diagram of the Experimental Apparatus Setup. Permeted from authers^35^.

The experimental setup used to study the kinetics of natural gas hydrate (NGH) formation in Bzc-modified Cu/Fe nanoparticle water-based systems is illustrated in Fig. 2^36^. The apparatus includes a durable, corrosion-resistant crystallizer with a 1.4 L capacity, capable of withstanding pressures up to 10 MPa. The system is equipped with precise temperature control, an accurate pressure gauge, and a high-resolution thermometer calibrated according to ASTM 1137 standards, allowing operation at temperatures as high as 673 K. Internal pressure is measured using a high-precision gauge (Model HD20V4T, Delta Ohm) with an accuracy of ± 0.02 MPa, while temperature readings are obtained using a thermometer with a ± 0.11 K margin of error. Thermal regulation is achieved through a water bath that circulates a glycol-water mixture around the crystallizer via a jacket system, controlled by a PID unit. A magnetic stirrer inside the crystallizer ensures homogeneous mixing of the contents. All sensor data are continuously recorded and transmitted to a computer through a control panel for real-time monitoring and analysis^36^.

### NGH formation procedure

This study focused on evaluating the influence of nanometals and the surfactant benzalkonium chloride (Bzc) on hydrate formation behavior. Two experimental approaches were employed: hydrate stability zone (HSZ) tests and kinetic studies. The HSZ experiments were designed to determine the pressure–temperature conditions under which the hydrate phase remains thermodynamically stable. The hydrate kinetics studies evaluated the total natural gas utilized in hydrate formation process, the formation rate, and the percentage of gas recovery during the dissociation phase. These experiments were performed for three materials NF, NFCu-Phy and NFCu-Ch. Several concentrations of each material were tested to find the optimal concentration under the best conditions. The experimental procedure commenced with the filling of a 400 cm³ crystallizer cell with distilled water. In some trials, water was pre-treated with varying concentrations of a promoter to evaluate its effect on hydrate formation. Prior to gas injection, the air above the liquid was evacuated, and the crystallizer was then charged with the target gas until a pressure of 6.5 MPa was reached. The internal temperature was gradually reduced from an initial value of 294.15 K in controlled steps using a precise thermal regulation system. At each step, the system was held for 60 min to ensure thermal and pressure equilibrium. Hydrate formation was identified by a noticeable and sudden pressure drop. Once formation was confirmed, the temperature was further decreased in small increments of 3–4 °C to comprehensively define the hydrate stability zone. For the kinetic experiments, initial conditions of temperature and pressure were selected to lie outside the hydrate stability region. The temperature was gradually reduced to trigger hydrate nucleation, which was marked by a sharp decline in pressure^36^. The induction time, which is the period before nucleation begins, along with pressure and temperature fluctuations over time, were meticulously monitored. Each kinetic experiment was repeated three times to ensure consistency, with pressure variations maintained within ± 0.1 MPa. After the hydrate formation experiments, the dissociation behavior of the formed hydrates was investigated using thermal stimulation, as per the established methodology^37^. During the dissociation experiments, the air bath surrounding the system was heated from the final hydrate formation temperature to 298.15 K under static (closed) conditions. As the temperature was increased, the system’s pressure initially rose gradually due to the thermal expansion of the gas phase. Once the temperature reached the equilibrium point for the prevailing pressure, the solid hydrate phase began to dissociate. This dissociation resulted in a sharp rise in pressure as the natural gas trapped within the hydrate structure was released. After the release of the trapped gas, both pressure and temperature stabilized, signaling the completion of the dissociation process. The final pressure was slightly higher than the initial value before dissociation, due to the thermal expansion of the released gas. This thermal stimulation technique provided a thorough examination of the hydrate dissociation behavior. The recorded pressure and temperature data yielded valuable insights into the dissociation dynamics under the given experimental conditions.

### Calculation method

The amount of natural gas consumed during hydrate formation, the rate at which hydrates were produced, the percentage of gas transformed into the hydrate phase, and the efficiency of natural gas release or recovery were assessed based on the recorded pressure and temperature data collected throughout the experiments.

#### The total moles of gas consumption

The total amount of gas moles consumed during the formation of hydrate or semi-clathrate hydrate was calculated according to Eq. (1)

1 $$\Delta {\mathrm{n}}_{ \downarrow } ,_{{\mathrm{H}}} = {\mathrm{V}}_{{\mathrm{g}}} \left( {\frac{{{\mathrm{P}}_{{\mathrm{i}}} }}{{{\mathrm{Z}}_{{\mathrm{i}}} {\mathrm{RT}}}}~ - ~~\frac{{{\mathrm{P}}_{{\mathrm{t}}} }}{{{\mathrm{Z}}_{{\mathrm{t}}} {\mathrm{RT}}}}} \right)$$

This equation involves several essential variables: the amount of natural gas consumed at a specific time (Δn↓,H), the gas volume within the crystallizer (Vg), the initial pressure at the start of hydrate formation (Pi), the initial compressibility factor (Zi), the ideal gas constant (R), the average temperature of the gas during the experiment (T), the reactor pressure at any given time (Pt), and the corresponding compressibility factor (Zt). Both the initial and instantaneous compressibility factors are calculated using the Pitzer correlation method, as detailed by Smith et al. (2001). The volume of natural gas consumed per unit volume of water is then calculated using a separate Eq. (2).

2 $${\mathrm{N}}_{{\mathrm{t}}} = ~\Delta {\mathrm{n}}_{ \downarrow } \left( {{\mathrm{mole}}/{\mathrm{mole}}} \right){\text{ }} = \Delta {\mathrm{n}}_{ \downarrow } ,_{{\mathrm{H}}} /{\text{ n}}_{{\mathrm{w}}}$$

n_w_ refer to the volume of the water in the crystallizer (mole).

#### Gas-to-hydrate conversion

The conversion rate of gas to hydrate (GH) at the end of each trial is evaluated by Eq. (3)^37^:

3 $${\text{GH }} = \Delta {\mathrm{n}}_{ \downarrow } ,_{{\mathrm{H}}} ~/{\text{ n}}_{{\mathrm{w}}} ~*\frac{{100}}{{{\mathrm{n}}_{{\mathrm{g}}} }}$$

Here, ng represents the initial number of moles of natural gas in the crystallizer at the beginning of each trial.

#### The rate of hydrate formation

The rate of NGH formation was calculated through a step-by-step numerical method, which involved monitoring the system’s changes at regular intervals, as described in Eq. (4)^38^.

4 $$\frac{{{\mathrm{dn}}}}{{dt}}~ = {\text{ }}\left( {\frac{{d\Delta {\mathrm{n}}_{ \downarrow } ,_{{\mathrm{H}}} }}{{{\mathrm{dt}}}}} \right)_{{\mathrm{t}}} ~ = \Delta {\mathrm{n}}_{ \downarrow } ,_{{\mathrm{H}}} \frac{{\Delta {\mathrm{n}}_{ \downarrow } ,_{{{\mathrm{H,}}}} {\mathrm{t}} + \Delta t - \Delta {\mathrm{n}}_{ \downarrow } ,_{{\mathrm{H}}} {\mathrm{t}}}}{{\Delta t}}$$

In this context, Δt denotes the time interval between two measurements, set at 30 s. The average rate of NGH formation was calculated at hourly intervals and presented graphically. The efficiency of NG recovery was then determined using Eq. (5)^39,40^:

5 $${\text{NG recovery }} = \Delta {\mathrm{n}}_{ \downarrow } ,_{{\mathrm{H}}} ~/{\text{ n}}_{{\mathrm{g}}} ~*{\text{ 1}}00$$

## Result and discussion

The natural gas plays a crucial role in today’s world, with modern methods increasingly aiming to store it in the form of hydrates. This study explores the use of nano particles metals hyperoid with cationic surfactant (Bzc) physically and chemically to form gas hydrates and study their effect on the amount of gas consumption; hydrate formation rate; water to hydrate conversion; induction time and gas storage capacity of natural gas hydrate.

###  The FTIR- spectra analysis of NFCu compounds

**Fig. 3:**
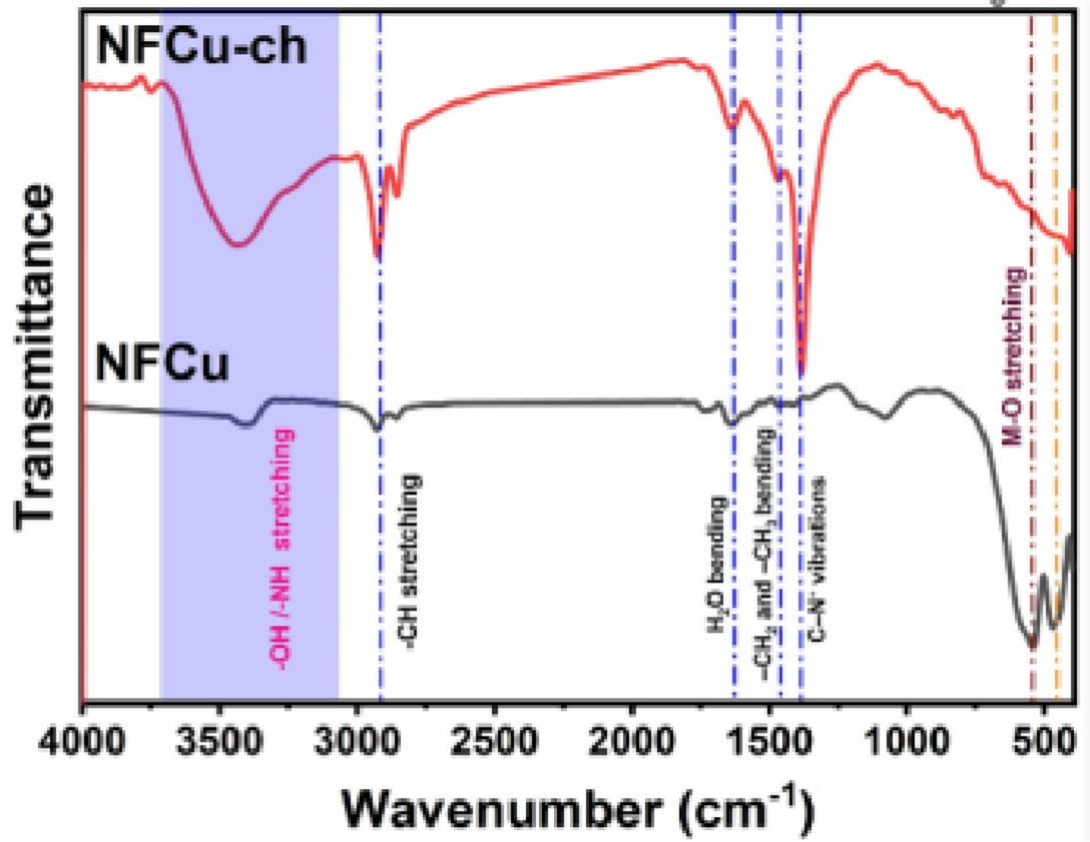
FTIR of The NFCu And NFCu- Ch.

The FTIR spectra of NFCu (CuFe_2_O_4_) and NFCu-Ch. (CuFe_2_O_4_ chemically attached Bzc) demonstrate distinct features corresponding to their composition as shown in Fig. 3. For NFCu (black curve), characteristic absorption bands for spinel ferrites were observed, particularly in the 500–600 cm^− 1^ region. These peaks correspond to metal-oxygen (M-O) vibrations, specifically Cu-O and Fe-O stretching modes, typical of spinel ferrite structures like CuFe_2_O_4_^31,41^. Additionally, a weak band around 1630 cm^− 1^ indicates bending vibrations of adsorbed water molecules (H_2_O), while the absence of significant peaks in the 3000–3500 cm^− 1^ region confirms the lack of organic functional groups. These observations are consistent with the reported spectra of spinel ferrites, as discussed previously^31,42^. In contrast, the FTIR spectrum of NFCu-Ch. (red curve) reveals notable changes due to the chemical attachment of benzalkonium chloride. A wide-ranging absorption band observed in the 3000–3500 cm⁻¹ region, highlighted in the graph, suggests the existence of O-H or N-H stretching vibrations from organic functional groups^42^, which are not observed in NF. The emergence of sharp peaks around 2900 cm^− 1^ corresponds to C-H stretching vibrations from the alkyl chains present in the benzalkonium chloride^41^.

New peaks at 1500 to 1600 cm^− 1^ range are also attributed to the aromatic ring vibrations or quaternary ammonium groups^43^. Despite these changes, the metal-oxygen (M-O) vibrations at the 500 to 600 cm^− 1^ regions extensively impact, indicating to the benzalkonium chloride which covered the core spinel ferrite structure of CuFe_2_O_4_ after the chemical modification. In summary, the FTIR analysis confirms the presence of CuFe_2_O_4_ spinel ferrite in NF, while NFCu-Ch. exhibits additional bands associated with benzalkonium chloride, confirming successful chemical attachment with the nano particles.

### The XRD analysis of NFCu compounds

**Fig. 4:**
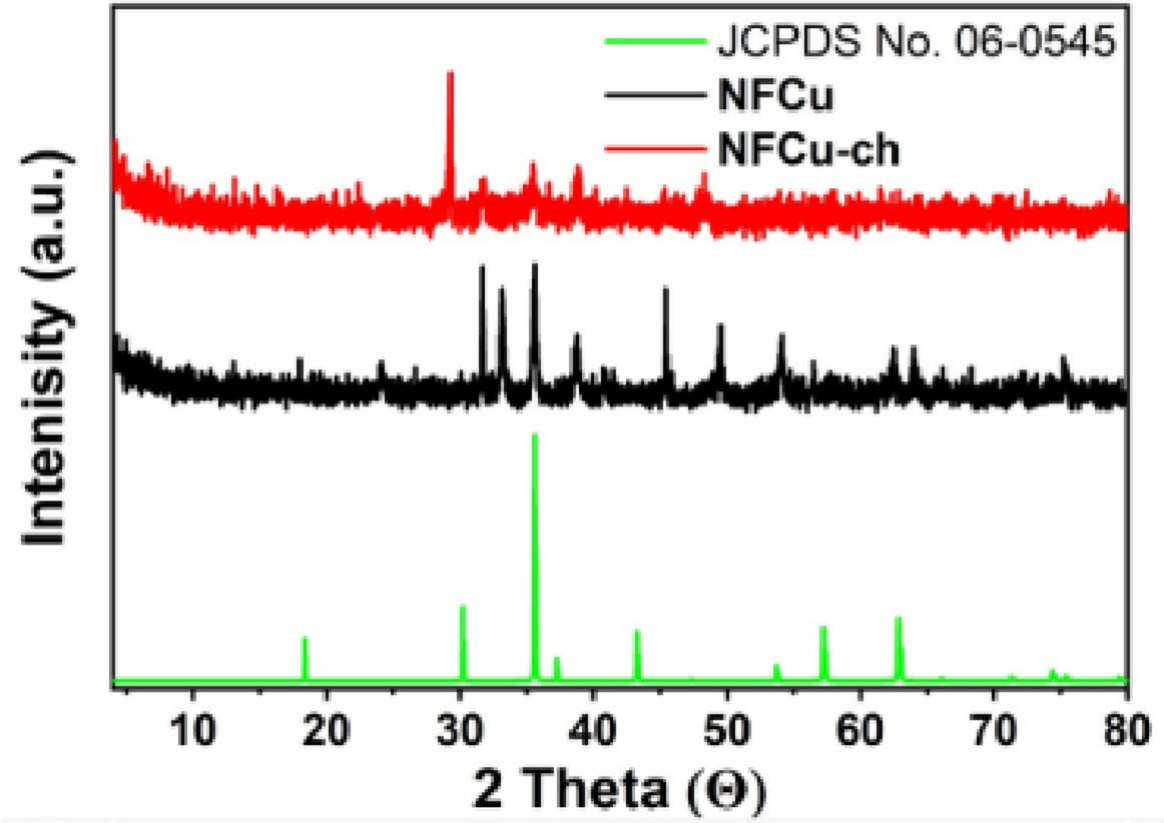
XRD of The NFCu And NFCu- Ch.

As displayed in Fig. 4 The XRD analysis of products NFCu and NFCu-Ch observes that the X-ray diffraction (XRD) profile of the CuFe_2_O_4_ at specific 2θ values that align with the corresponding diffraction planes listed in JCPDS No. 06–0545. The key diffraction peaks observed at 31.67° (200), 33.16° (103), 35.60° (211), 38.76° (202), 40.89° (004), 45.43° (220), 49.49° (312), 54.11° (105), 56.49° (303), 62.47° (224), 64.01° (400), and 75.31° (206) confirm the crystalline nature of CuFe_2_O_4_^44,45^. The absence of any additional peaks suggests high phase purity, with no detectable secondary phases or impurities present in the sample, which also supported successful synthesis of the prepared material.

After the insertion of Bzc onto the CuFe_2_O_4_ surface, the XRD pattern reveals a significant change, with the disappearance of the lower intensity peaks. Only peaks at 29.93°, 32.07°, 35.3°, 37.9°, and 48.1929° remain. The shifting of those peaks to lower diffraction angles suggests that the presence of Bzc has induced a transition to an amorphous or less ordered state, affecting the crystallinity of CuFe_2_O_4_. This could be resulting from Bzc molecules adhering to the nanoparticle surfaces., disrupting the crystalline structure and lowering the intensity of the characteristic peaks. The crystalline size (Dc) of CuFe_2_O_4_ nanoparticles was estimated by using the Scherrer Eq. 6:

6 $$Dc=\frac{K\lambda}{\beta cos\varTheta}$$

Where β is the full width at half maximum (FWHM) of the diffraction peak, λ is the X-ray wavelength, and K is the shape factor (typically 0.9). As perceived from data in Table 3, For CuFe2O4, the particle size was calculated to be 14.59 nm at the peak of 31.67°. In comparison, the Bzc/CuFe2O4 sample showed an increased particle size of 104.15 nm at a lower shifted angle of 29.93° due to the bulky, hydrophobic bi-tailed structure of Bzc, which promotes agglomeration and enlarges the particle size^46^.

**Table 3:** XRD Crystallographic Parameters of Products NFCu and NFCu - Ch.

Sample	FWHM (β)	Diffraction peak (Θ)	Particle diameter (nm)
NFCu	0.0984	31.67	14.59035
NFCu - Ch.	0.1574	29.93	104.1486

###  the scanning electron microscopy NFCu compounds

**Fig. 5:**
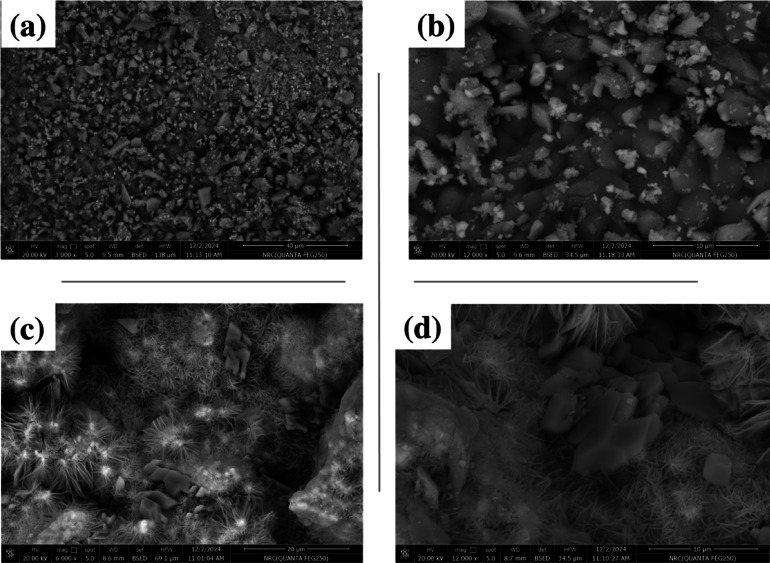
SEM Images of The Synthesized Nanoparticles; (a) and (B) represent NFCu, (C) and (D) Depict NFCu- Ch.

The SEM images of CuFe_2_O_4_ and CuFe_2_O_4_/Bzc revealed distinct morphological characteristics that highlight their functionality. As shown in Fig. 5 The CuFe_2_O_4_ images (a) and (b) demonstrate regular and relatively uniform shapes. At lower magnification, the particles appear as dense aggregates with an irregular granular structure, while at higher magnification, the particles exhibit well-defined boundaries and a crystalline nature. However, morphology lacks a network-like framework, limiting the presence of structural cavities for enhanced gas adsorption. In contrast, the SEM images of CuFe_2_O_4_/Bzc (c) and (d) showed a flower-shaped network induced by the Bzc surfactant. This intricate structure comprises radiating thin sheets and needle-like projections, with CuFe_2_O_4_ particles deposited across the framework. This deposition increases the surface area and creates well-defined cavities, making the material highly absorbent for natural gas. The flower-like network provides hierarchical cavities ideal for methane adsorption and storage, significantly enhancing the functional capabilities of the composite material.

### **The Brunauer–Emmett–Teller analysis NFCu compounds**

**Fig. 6:**
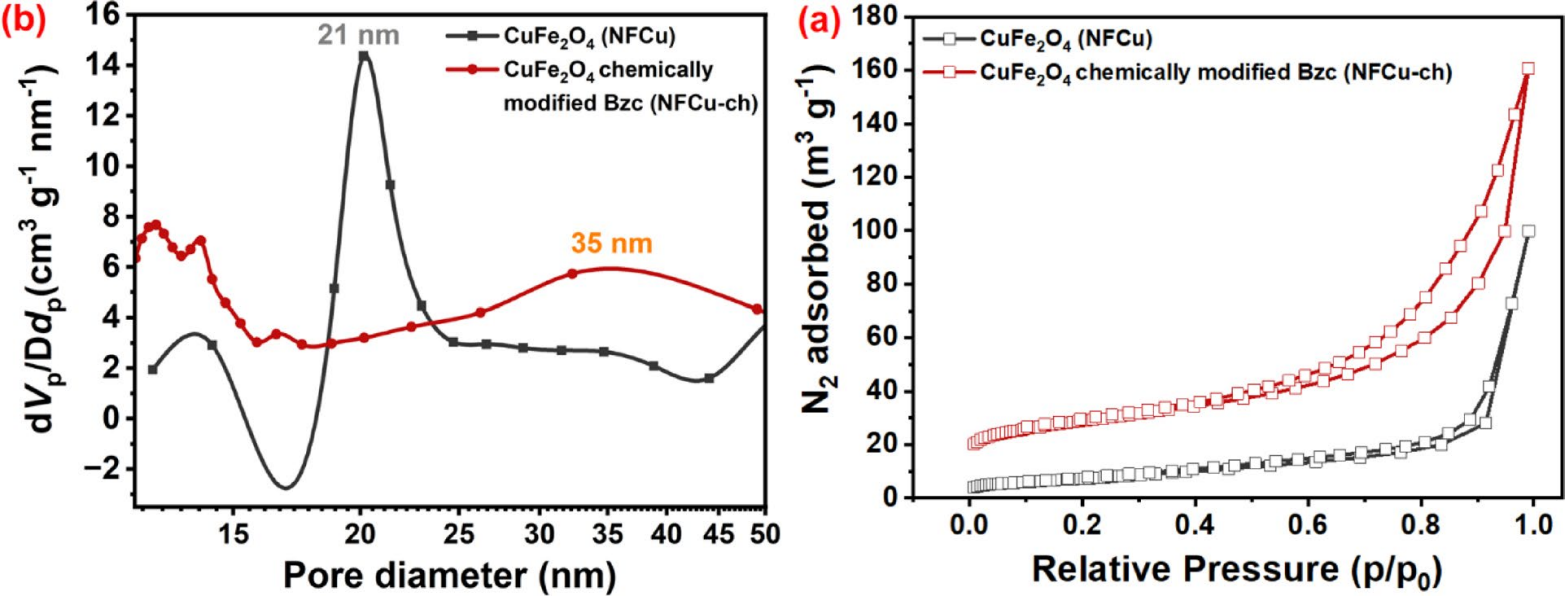
**a** Nitrogen Adsorption–Desorption Isotherms (BET) and **b** BJH Pore Size Distribution for NFCu and NFCu-ch.

Figure 6 (a) and (b) provide comprehensive insight into the textural properties of pristine CuFe_2_O_4_ (NFCu) and chemically modified CuFe_2_O_4_ (NFCu-ch). In Fig. 6 (a), the nitrogen adsorption–desorption isotherms of both samples exhibit type IV behavior with H3-type hysteresis loops, indicating that the presence of mesopores. Notably, NFCu-ch shows a significantly greater nitrogen adsorption volume than the NFCu, implying (63.8 m_2_/g) a substantial increase in the surface area (127.4 m_2_/g) due to its chemical modification.

This enhancement can be attributed to the introduction with the surface-active agent molecules or structural reorganization, which generates more accessible mesoporous regions, like trends observed in other CTAB- or surfactant-assisted syntheses^47–49^. Figure 6 (b) presents the BJH pore size distribution, where NFCu exhibits a sharp and narrow peak centered around 21 nm, indicative of uniform mesoporous structures. In contrast, as shown in Table 4 the NFCu-ch displays a broader distribution centered around 35 nm, suggesting an increase in both average pore size and total pore volume. This shift is consistent with pore-expanding effects observed in related to the CuFe-based systems prepared by surfactants or templating agents^50,51^.

**Table 4:** BET Parameters of Products NFCu and NFCu - Ch.

Sample	S_BET,_ m_2_/g	Pore diameter (nm)
NFCu	63.8	21
NFCu - Ch.	127.4	35

Such structural evolution is favorable for improving the surface-related functionalities such as catalytic activity and absorption ability^52,53^. Overall, the BET data confirm that chemical modification of CuFe_2_O_4_ effectively enhances its mesoporosity and surface area, key attributes for boosting its performance across adsorption and absorption substrate applications.

This substantial increase in specific surface area, rising from 63.8 to 127.4 m^2^/g, plays a decisive role in boosting hydrate formation performance. By providing a higher density of active nucleation sites at the solid-liquid interface, the modified nanoparticles lower the energy barrier for hydrate embryo stabilization. This direct correlation explains the observed kinetic improvements, specifically the reduction in induction time and the higher total gas uptake, as the expanded surface area facilitates more efficient mass transfer and gas-water contact. Consequently, these enhanced textural properties are key attributes for boosting performance across adsorption and hydrate-based gas storage applications.

### Stability of the prepared solution NFCu compounds

**Fig. 7:**
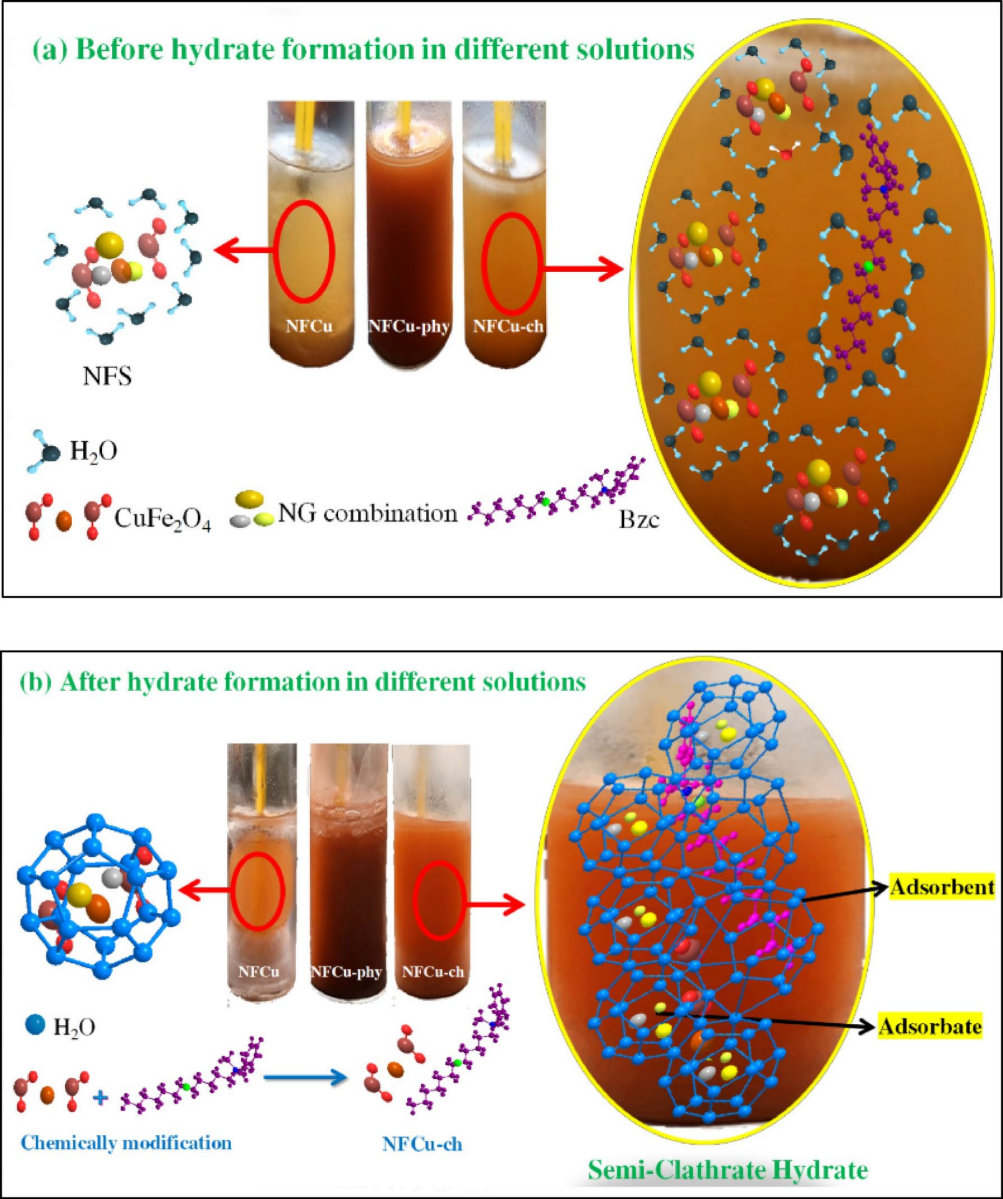
Represent the Stability And the Mechanism of Reaction Befor and After Hydrate Formation Process for NFCu,. NFCu-Phy. And NFCu-ch.

The chemical modification of the CuFe_2_O_4_ nanoparticles with benzalkonium chloride (Bzc) (NFCu-Ch.) was investigated for its ability to enhance natural gas hydrate (NGH) formation, as depicted in Fig. 7. The modified nanofluid (NFCu-Ch.) demonstrated significantly improved stability compared to the individual CuFe_2_O_4_​ nanofluid (NFCu) or nanofluids containing only Bzc (NFCu-Phy.) without prior reaction. When the unmodified CuFe_2_O_4_ nanoparticles were introduced into deionized water, they tended to aggregate into larger clusters, even under ultrasonic agitation, ultimately leading to precipitation. Conversely, the aggregation reduced in the presence of adsorbed Bzc molecules on the surface of the CuFe_2_O_4_ ​ nanoparticles. While small clusters still formed, they remained dispersed as nanoscale aggregates (100 nm) at case of physical mixture. meanwhile, in the case of chemical modification of ferrite were stabilized by Bzc.

### Kinetics of NGH

The experimental procedures were performed under regulated conditions, keeping the starting pressure of 6.5 MPa and the temperature constant of 273.15 K. Three products; NF and NFCu-Phy. supplemented physically blending with various concentrations of Bzc, and NFCu-Ch. were investigated as potential kinetic enhancers for NGH formation The experimental details are summarized in Table 5. The duration of the experiments was 120 min from the beginning of hydrate formation. Aqueous solutions of NFCu were prepared at multiple concentrations (0.01, 0.02, 0.1 and 1 wt%) in pure water to determine the optimal concentration.

**Table 5:** Induction Time, Final gas Consumption, Gas Conversion to Hydrate and NG Recovery During Hydrate Formation at Different Concentrations of Unmodified CuFe_2_O_4_ (NFCu), Physically Blended CuFe_2_O_4_/Bzc (NFCu-Phy.) and Chemically Modified CuFe_2_O_4_-Bzc (NFCu- Ch.).

Aqueous system	EXP. No.	Additives Conc., wt %	Induction Time, Min.	No. of mol gas/mol water	Gas to Hydrate Conversion, %	NG Recovery, % (Dissociation)
NG + NFCu	1. 2. 3. 4.	0.02 0.01 0.1 1	12 15 18 24	0.12 0.10 0.07 0.08	18.43 18.53 17.30 18.49	81.52 79.61 80.44 79.35
NG + NFCu - Phy.	1. 2. 3. 4.	0.03 ppm 0.05 ppm 0.10 ppm 0.15 ppm	8 6 10 12	0.22 0.26 0.24 0.19	35.24 33.41 34.99 35.54	92.10 93.35 91.41 92.38
NG + NFCu - Ch.	1. 2. 3. 4.	0.001 0.003 0.005 0.01	10 6 5 15	0.32 0.33 0.35 0.20	45.15 44.77 46.10 45.14	97.30 97.44 97.86 95.57

**Fig. 8:**
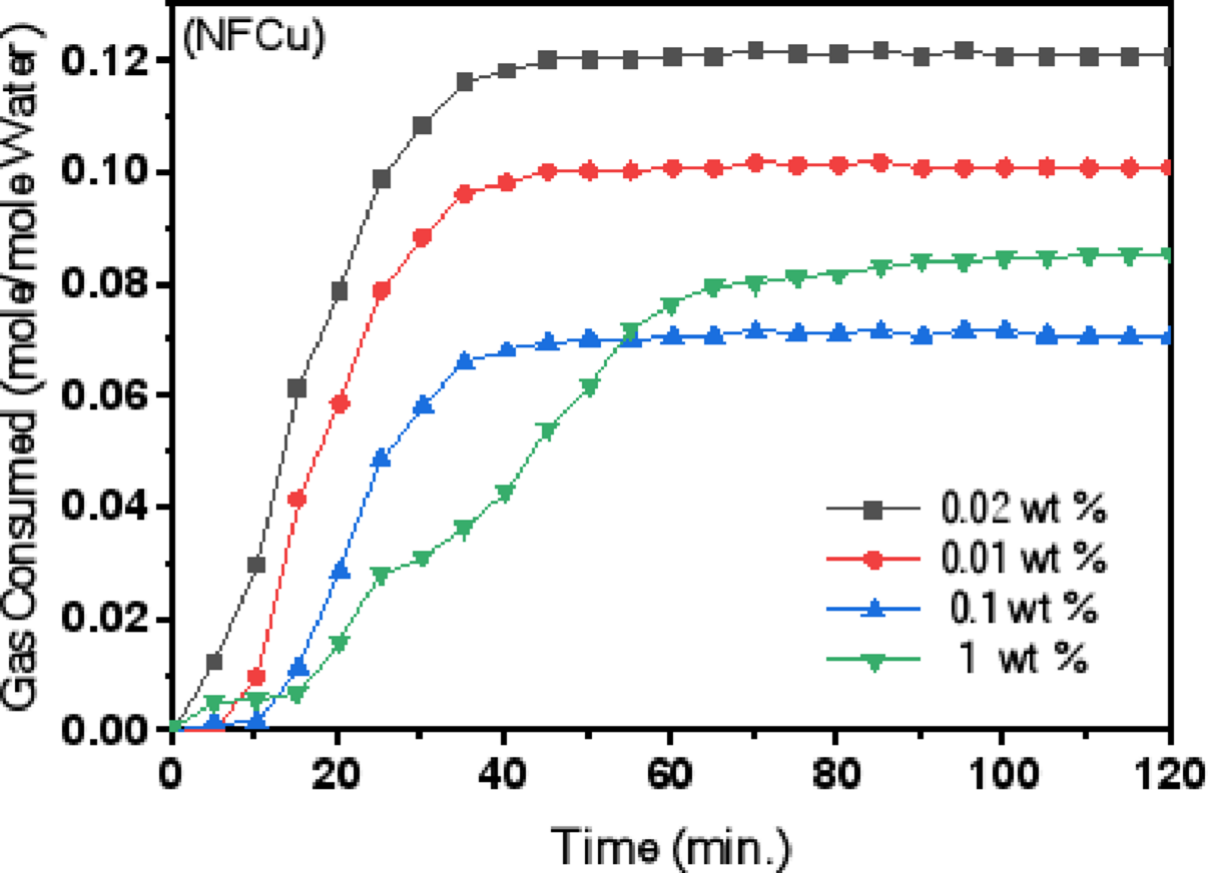
Gas Consumption Against Time for NFCu at Various Concentrations of CuFe_2_​O_4_ ​.

This optimum concentration was then combined with different dosages of Bzc (0.03, 0.05, 0.10 and 0.15 wt%) to identify the most effective concentration for promoting hydrate formation. Finally, NFCu-Ch. was tested at various weight percentages (0.001, 0.003, 0.005 and 0.01 wt%) to evaluate its performance under the same experimental conditions. The products were evaluated based on phase equilibrium, induction time, hydrate formation rate, and natural gas recovery. In Fig. 8 The analysis of gas consumption over time for different concentrations of Cu/Fe_2​_O_4_ (NF) revealed that the 0.02 wt% is the optimal concentration, achieving the highest gas consumption (0.12 mol/m mole water) with a relatively short induction time (time to initiate significant gas consumption) of around 10 min. In comparison, 0.01 wt% shows the shortest induction time (5 min) but reaches a slightly lower gas consumption (0.10 mol/m mole water), making it a suitable alternative for applications requiring faster reaction initiation. The higher concentrations, such as 0.1 wt% and 1 wt%, demonstrate longer induction times (18 and 24 min, respectively) and lower gas consumption values (0.07 and 0.06 mol/m mole water), indicating that the increase of the concentration beyond 0.02 wt% does not enhance the performance.

**Fig. 9:**
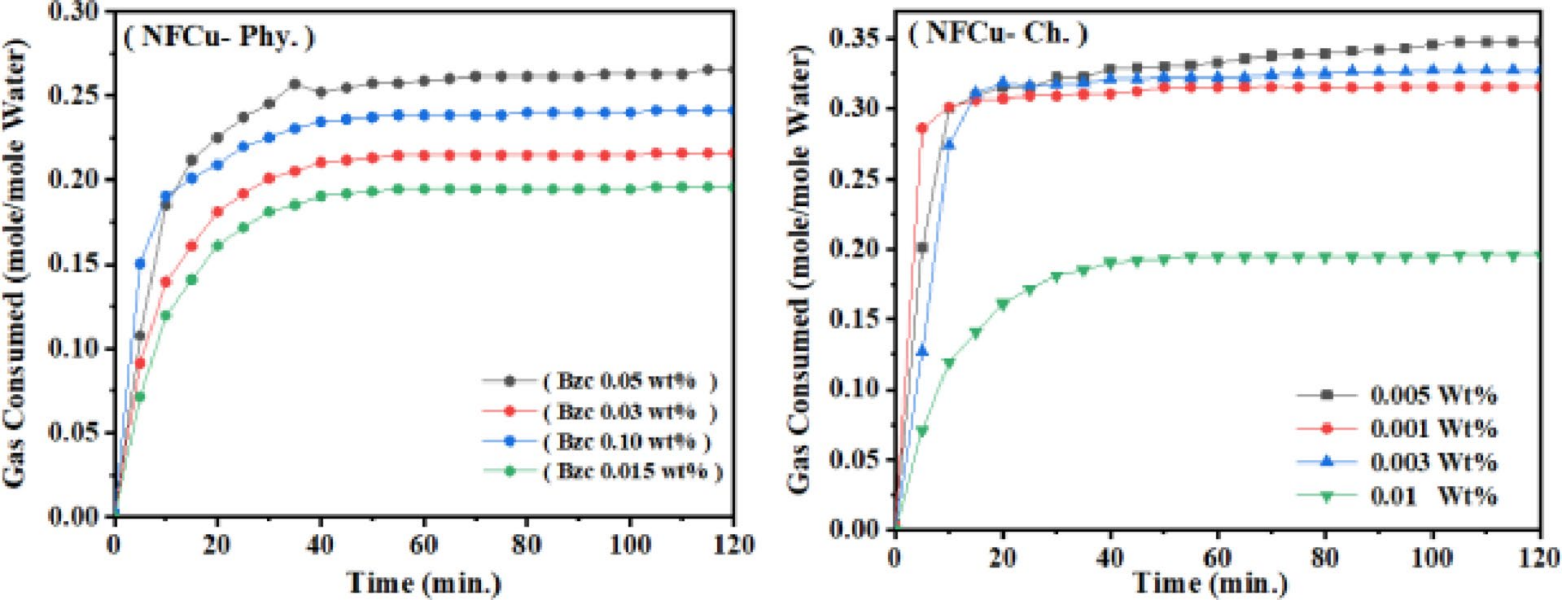
Gas Consumption Against Time for (a) NFCu- Phy. Supplemented with Various Concentrations of Bzc and (B) Different Concentrations of NFCu- Ch.

**Fig. 10:**
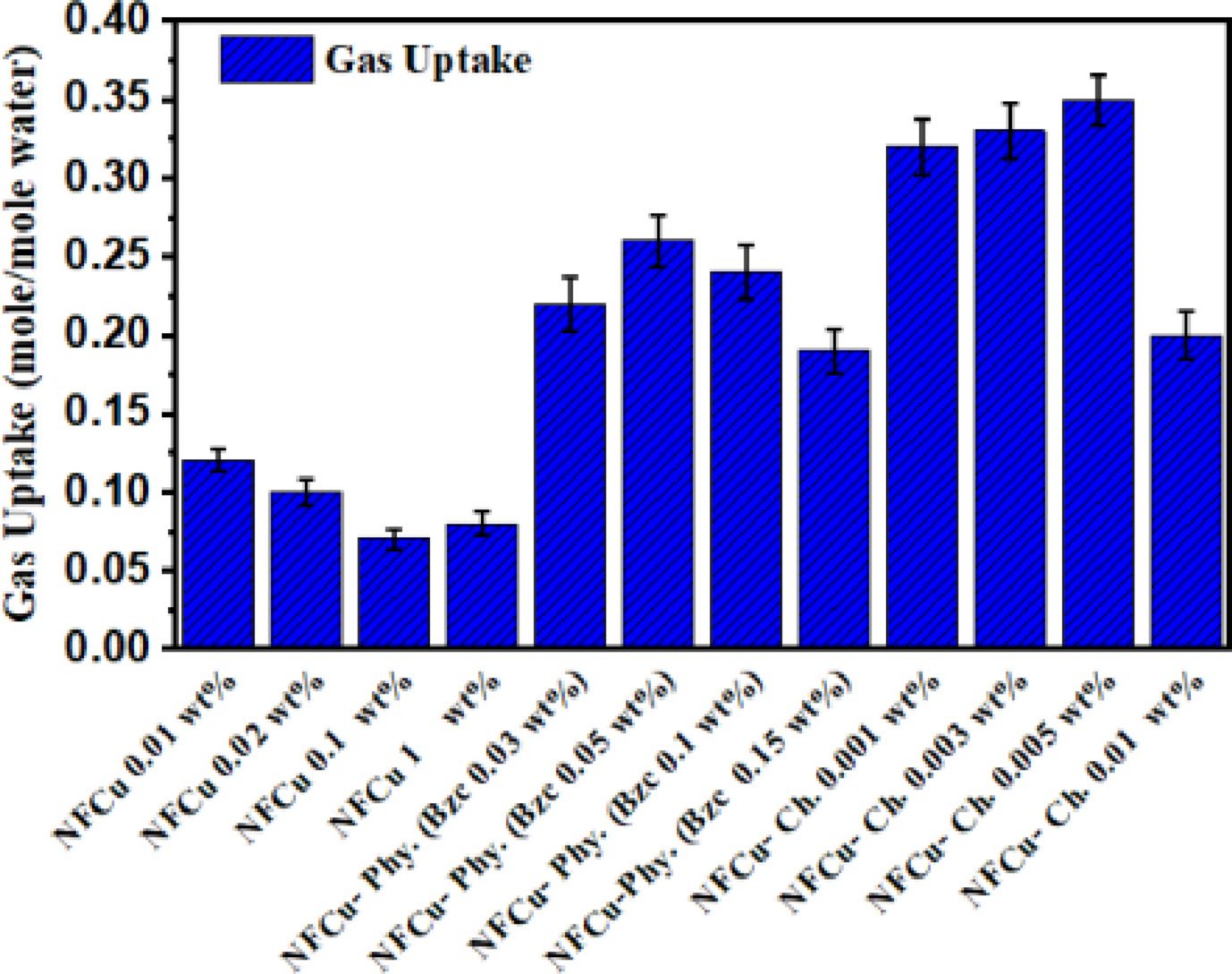
Variation of Total Gas Consumed at Different Concentrations of NFCu, NFCu- Phy. And NFCu- Ch Aqueous Solution.

Therefore, 0.02 wt% is the best choice for maximizing gas consumption, balancing both efficiency and induction time. As shown in Fig. 9. the gas consumption for the optimum concentration of NFCu (0.02 wt%) with different Bzc concentrations, exhibited at 0.05 wt% the Bzc achieves the highest gas consumption (0.26 mol/m mole water) with a short induction time, making it the most effective concentration. While the gas consumption decreases, with 0.10 wt% and 0.03 wt% showing moderate values (0.23 and 0.21 mol/mole water, respectively). The 0.15 wt% concentration exhibits the lowest gas consumption (0.20 mol/m mole water) and the longest induction time. Similarly, in Fig. 9 At 0.005 wt%, the induction time is approximately 5 min, and the gas consumption stabilizes at around 0.35 mol/mole water. Moreover, for 0.003 wt%, the induction time was also about 6 min, with a final gas consumption of approximately 0.34 mol/mole water. For 0.001 wt%, the induction time was slightly longer, around 10 min, and the gas consumption reached 0.32 mol/mole water. In contrast, at the highest concentration of 0.01 wt%, the induction time increased to about 15 min, and the final gas consumption significantly droped to approximately 0.20 mol/mole water. These results indicated that the low concentrations (0.001–0.005 wt%) of NFCu-Ch. promote high gas consumption efficiency and short induction times, while excessive concentration (0.01 wt%) was negative impact on both gas consumption and induction time. As exposed in Fig. 10, the NFCu-Ch. exhibited more storage capacity than the NFCu and NFCu-Phy. blended physically with Bzc solution.

### NGH induction time

**Fig. 11:**
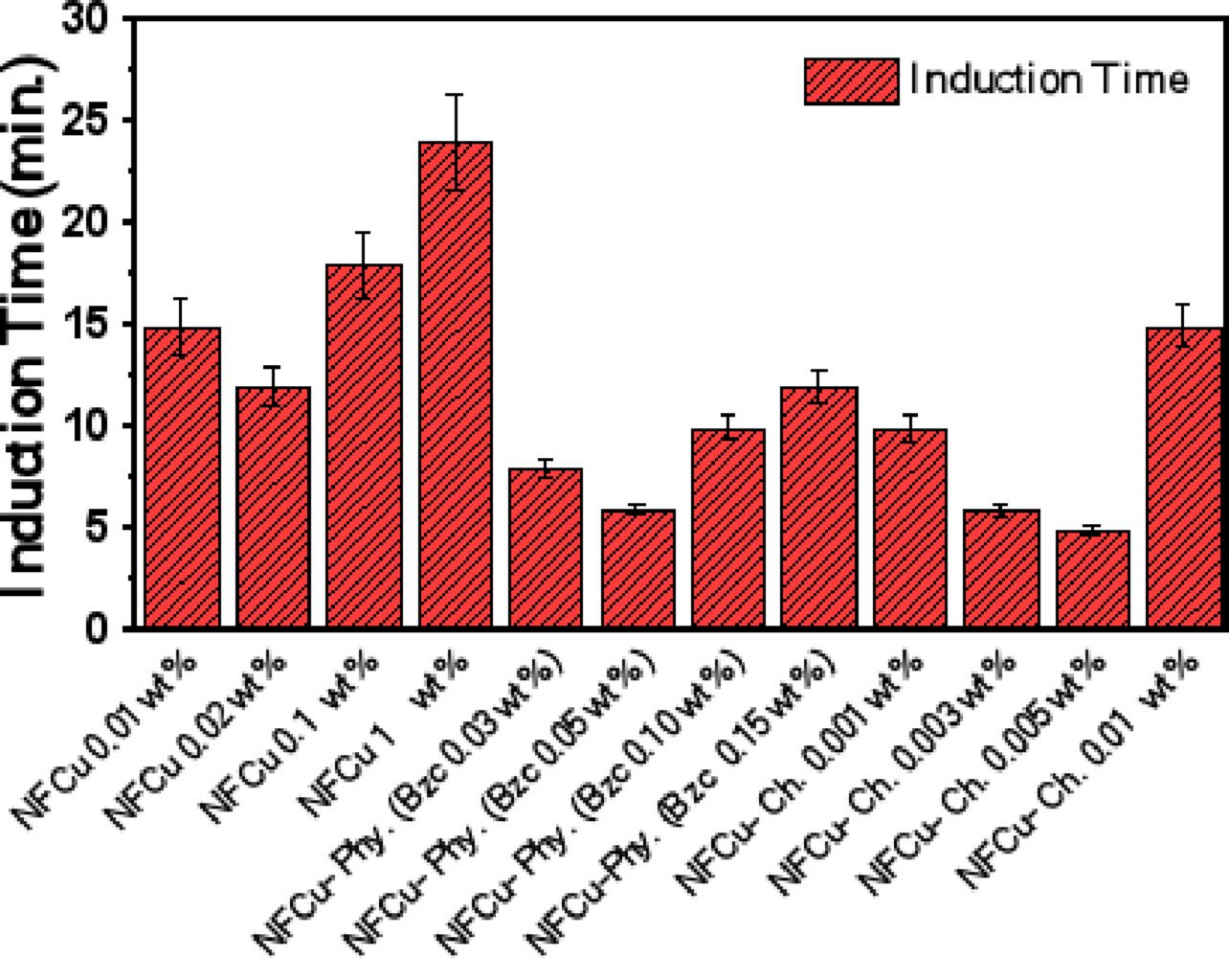
Variation of Induction Time at Different Concentrations of NFCu, NFCu- Phy. and NFCu- Ch. Aqueous Solution.

Induction time is a critical factor in the process of forming gas hydrates, representing the interval between the establishment of experimental conditions, such as temperature and pressure, and the visible initiation of nucleation and growth of hydrate. This phase reflects the time needed for the structure to achieve the required Metastable state and for the forming first hydrate crystals. Various factors, including the applied temperature and pressure, the existence of modifiers that either enhance hydrate formation, system mixing, and the obtainability of crystal initiation points, can significantly affect induction time. By studying and quantifying this period, valuable insights can be gained into the underlying kinetics and mechanisms of the process of forming hydrate, enabling the optimization of processes involving gas hydrates. Assessing and evaluating the duration before initiation can offer key details about the formation dynamics and reaction pathways of hydrates, which is essential for gaining insights into and improving the efficiency of gas hydrate operations^54^. As shown in Fig. 11, the optimal concentration of NF is 0.02 wt% of CuFe₂O₄, exhibited induction time 12 min for the appearance of nucleation sites. When it was blended physically with Bzc, the induction time decreases to less than 12 min. Furthermore, the adding of Bzc enhances the stability of the nano-metal solution to remain more uniformly suspended in the bulk, which, in turn, optimizes both the induction time and the total hydrate growth during NGH formation^35^. On the other hand, (NFCu-Ch.), which was chemically modified between Bzc and the cupper ferrite, pronounced high significant decrease in the induction time (5,6 and 10 min.) against low concentrations (5,3 and 1 * 10^− 3^ wt%). Whereas the physically modified nano particles by Bzc exhibited 15 min. at high concentration.

### The rate of NGH formation

**Fig. 12:**
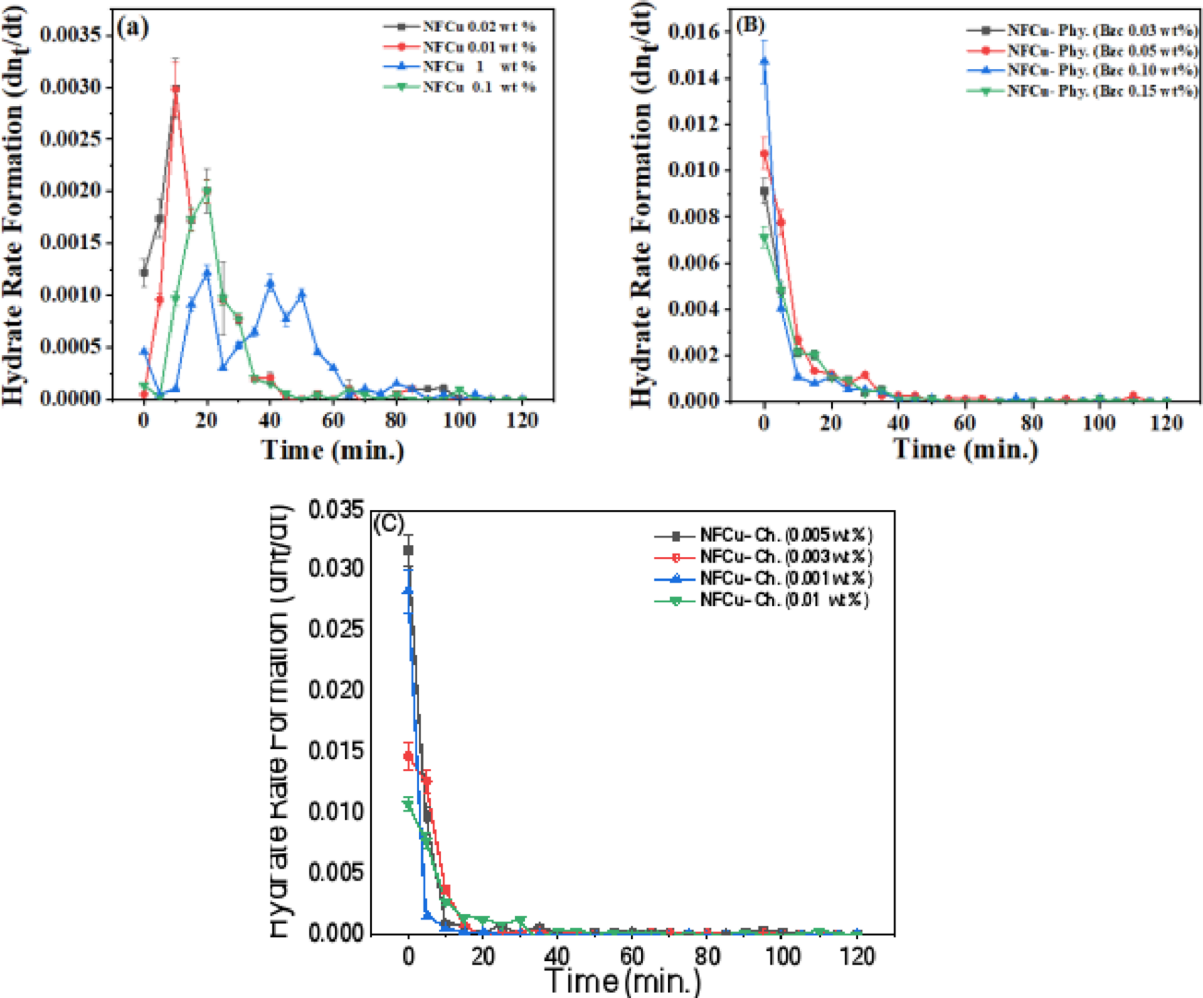
NGH Rate Formation at Different Concentration of (a) NFCu, (B) NFCu- Phy. and (C) NFCu- Ch. Aqueous Solution.

The rate of Natural Gas Hydrate (NGH) formation is a critical parameter in understanding the hydrate development, which occurs under specific thermodynamic conditions. This rate is affected by several interrelated factors, involving the temperature and pressure of the environment, the concentration of gas and water, the presence of sites of nucleation which facilitate the initial formation of the hydrate crystals and the character of the used promoter in the hydrate formation process and its concentration. The kinetics of NGH formation described the dynamic equilibrium between the hydrate formation and dissociation by using mathematical. This equilibrium is sensitive to the concentrations. As illustrated in Fig. 12, the hydrate rate formation was calculated to show the influence of the promoters on the hydrate system and their efficiency. So that the Fig. 12 (a) shows the hydrate formation rate across different concentration levels of NF, which exhibited the highest formation rate at 0.01, 0.02, and 0.1 wt %. Although the high formation rate achieved by NF, but the stability of the hydrate nuclei was inconsistence as shown in Fig. 12 (a). this inconsistence may be due to the complex. Additionally, the experimental factors such as localized inhomogeneities, variability in the gas solubility, or differences in heat and mass transfer could also contribute to the observed variations.

Furthermore, Fig. 12 (b) illustrates the synergistic effect of physical combining of NF (0.02) with Bzc at different concentrations. The highest hydrate formation rate was observed at 1000 ppm of Bzc, followed by 500 ppm, 300 ppm, and the minimum rate was at 1500 ppm. From the point of view, the physical combination of NFCu-Phy. enhances the hydrate formation and decreases the variation of addition NF. This find may be due to the Bzc modifies the interfacial properties and enhances the dispersion stability of the nanoparticles. The maximum enhancement development in the rate of hydrate formation was depicted by the chemically interaction between Cu/F_2_O_4_ and Bzc. As shown in Fig. 12 (C), the highest initial rate was observed at 0.001, followed by 0.005, 0.003, and the lowest initial rate at 0.01 ppm. From the obtained data, it was found that the chemically binding of Bzc with Cu/F_2_O_4_ which named NFCu-Ch. increases the rate of hydrate formation more than the physical blending or the individual nanoparticles due to the modification of hydrate morphology as shown previously^50^. The modification of morphology may be helped to distribute the gas molecules regularly on the surface of the promoters and its internal sites and cavities which causing the increase of average hydrate formation and regularity of the growth formation.

### Natural gas liberation during the dissociation stage

**Fig. 13:**
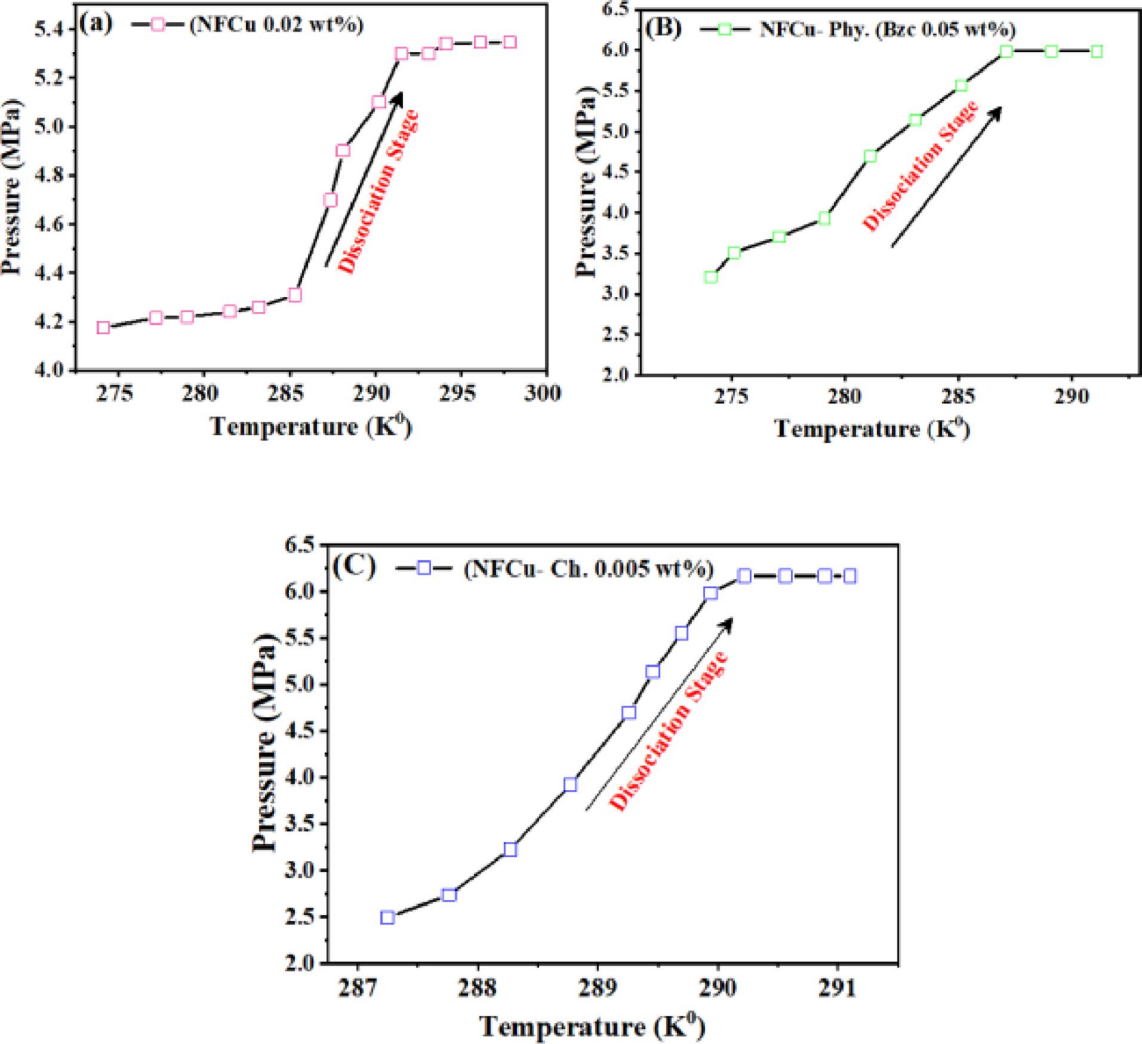
Pressure–Temperature Plot During Hydrate Dissociation Stage at **a** NFCu, **B** NFCu- Phy. and **C** NFCu- Ch.

The dissociation characteristics of natural gas hydrates (NGHs) were examined with three different additives-NFCu, NFCu-Phs., and NFCu-Ch. using pressure and temperature (P-T) analysis. The system incorporating nano-metal materials showed a noticeable shift in the slope of the dissociation graph in Fig. 13, indicating a possible phase change or modification in the hydrate’s internal structure^55,56^. The Fig. 13 illustrates the dissociation behavior of hydrates under different experimental conditions and cleared the variations in the dissociation rates and system recovery of NGH. Figure 13 (a) shows a gradual increase in data points before the dissociation stage, suggesting a slower hydrate dissociation process. The dissociation curve exhibits a relatively lower slope, indicating that the dissociation occurs over an extended period or under milder external conditions, such as lower temperatures or gradual pressure reduction. This behavior suggests the presence of a less aggressive dissociation mechanism, potentially influenced by a lower heat flux.

From the obtained data, it was observed that the experiment began at a pressure of 6.5 MPa, and throughout the dissociation stage, the pressure decays up to 5.3 MPa and did not return to the initial pressure 6.5 MPa (81.51% natural gas recovery). The variation between the initial and final pressures during the dissociation process, means that the nano particles without modification trapped some of hydrate (named as latent hydrate). In contrast, Fig. 13 (b) shows a sharper dissociation curve with a steeper slope, indicate to the fast transition from stability to the dissociation phase. This rapid behavior can be attributed to higher temperatures; abrupt pressure drops as a result of presence physically complex of Bzc with nano particles CuFe_2_O_4_ (NFCu-Phy.). From the resulting data, it is evident that the Bzc enhances the solution’s ability to recover the initiating pressure more than at case of the individual nanoparticles (NF) with the final pressure reaching 5.99 MPa (90.7% recovery of natural gas). The near close pressure recovery in this case (5.99 to 6.5 MPa) may be due to the physical charge energy of Bzc, which led to decrease the amount of the trapped hydrate in the complex (NFCu-Phy.). Furthermore, Fig. 13 (c) displays a more linear progression of dissociation, characterized by a steady and consistent slope. This behavior suggests a controlled dissociation process, possibly achieved through heating program or pressure adjustment. The linearity indicates to uniform dissociation rate, with the natural gas recovery, 95% of the starting pressure, demonstrating significantly better pressure recovery compared to the previous two cases. The variations in dissociation behavior are likely due to the chemical reactions occurring between the Bzc and the nanoparticles which led to increase of the surface charge energy. This modification and charge of surface energy enhances the natural gas recovery up to 95%^57^. The results provide valuable insights into how various additives impact the stability and the behavior of natural gas hydrates during dissociation. This understanding is essential for improving gas storage, transportation, and extraction methods, as well as for devising effective strategies to manage hydrates efficiently.

### Mechanism of hydrate formation by the nanoparticles and their derivatives

The modification of CuFe₂O₄ nanoparticles with Bzc physically or chemically significantly enhances their performance toward NGH formation compared to unmodified CuFe₂O₄. The addition of Bzc, a quaternary ammonium surfactant, improves the nanoparticle dispersion in the aqueous systems and increases its interaction with hydrates and gas molecules. The molecular analysis revealed that the Bzc-modification of CuFe₂O₄ enhances the electron transfer capabilities, as evidenced by altered HOMO-LUMO orbital distributions. Furthermore, the solvent-accessible surface area of the modification nanoparticles is higher than the unmodified CuFe₂O₄, providing more active sites for gas adsorption and hydrate nucleation. Experimental results show that the Bzc-physically modification of CuFe₂O₄ reduces induction time and promotes faster hydrate formation rates. These results may be traced back to the surfactant’s ability to lower surface tension^55^ and improve gas–liquid interaction. otherwise, unmodified CuFe₂O₄ shows limited efficacy due to agglomeration and fewer active sites for hydrate nucleation. This comparison cleared the modification role to optimize and enhance the nanoparticle performance for gas hydrate applications, making the Bzc-chemically modified of CuFe₂O₄ is superior candidate for hydrate-based gas storage among the physical modification on individual nanoparticles and transportation technologies. As shown in Tables 6 and 7, the distribution of electronic charge in CuFe₂O₄ and Bzc -physically and chemically modification of CuFe₂O₄ proved that the modification has a function in the electronic properties of the nanoparticles, which directly influence their performance in natural gas hydrate formation.

**Table 6:** Charge Distribution for NFCu and NFCu - Ch. According to Extended Huckel Theory.

NFCu		NFCu - Ch.					
Atom Number	Charge Value (eV)	Atom Number	Charge Value (eV)	Atom Number	Charge Value (eV)	Atom Number	Charge Value (eV)
Fe(1)	−3.56074	Fe (1)	−3.56284	C (10)	0.12062	C (21)	0.169728
O(2)	1.9601	O (2)	0.430854	C (11)	0.157451	C (22)	0.00486048
O(3)	1.71147	O (3)	0.263581	C (12)	0.176958	C (23)	0.176616
Fe(4)	−3.53428	Fe (4)	−3.54059	C (13)	0.180229	C (24)	0.284912
O(5)	1.30773	O (5)	−0.476652	C (10)	0.12062	C (28)	0.0627337
O(6)	2.38971	O (6)	−0.390703	C (11)	0.157451	C (29)	0.0511561
Cu(7)	0.725998	Cu (7)	−0.0351799	C (12)	0.176958	Cl (30)	3.31153
		C (8)	0.193632	C (13)	0.180229	H (31)	0.0293342
		C (9)	0.101785	C (14)	0.167861	H (32)	0.0253795
		C (10)	0.12062	C (15)	0.138717	H (33)	0.034855
		C (11)	0.157451	C (16)	0.0977894	H (34)	0.0361526
		C (12)	0.176958	C (17)	0.0527964	H (35)	0.0287632
		C (13)	0.180229	C (18)	0.0144355	H (36)	0.0289161
		C (14)	0.167861	C (19)	−0.10108	H (37)	0.0277868
		C (15)	0.138717	N (20)	0.8797	H (38)	0.0277781
		C (16)	0.0977894	C (21)	0.169728	H (39)	0.0278389
		C (17)	0.0527964	C (22)	0.00486048	H (40)	0.0278
		C (18)	0.0144355	C (23)	0.176616	H (41)	0.027797
		C (19)	−0.10108	C (24)	0.284912	H (42)	0.0277771
		N (20)	0.8797	C (25)	0.241569	H (43)	0.0278
		C (21)	0.169728	C (26)	0.0710687	H (44)	0.0277938
		C (22)	0.00486048	C (27)	0.0550728	H (45)	0.0278461
		C (23)	0.176616	C (14)	0.167861	H (46)	0.0278234
		C (24)	0.284912	C (15)	0.138717	H (47)	0.0279085
		C (25)	0.241569	C (16)	0.0977894	H(48)	0.0279008
		C (26)	0.0710687	C (17)	0.0527964	H (49)	0.0280862
		C (27)	0.0550728	C (18)	0.0144355	H (50)	0.028064
		C (28)	0.0627337	C (19)	−0.10108	H (51)	0.0290624
		C (29)	0.0511561	N (20)	0.8797	H (52)	0.0290559
		Cl (30)	3.31153	C (21)	0.169728	H (53)	0.0459209
		H (31)	0.0293342	C (22)	0.00486048	H (54)	0.0390586
		H (32)	0.0253795	C (23)	0.176616	H (55)	0.0390369
		H (33)	0.034855	C (24)	0.284912	H (56)	0.188468
		H (34)	0.0361526	C (25)	0.241569	H (57)	0.0590152
		Fe (1)	−3.56284	C (26)	0.0710687	H (58)	0.0510046
		O (2)	0.430854	C (27)	0.0550728	H (59)	0.0629702
		O (3)	0.263581	C (14)	0.167861	H (60)	0.0532741
		Fe (4)	−3.54059	C (15)	0.138717	H (61)	0.0599819
		O (5)	−0.476652	C (16)	0.0977894	H (62)	0.0425848
		O (6)	−0.390703	C (17)	0.0527964	H (63)	0.04955
		Cu (7)	−0.0351799	C (18)	0.0144355	H (64)	0.232689
		C (8)	0.193632	C (19)	−0.10108	H (65)	0.0277048
		C (9)	0.101785	N (20)	0.8797	H (66)	0.0441686
						H (67)	0.0507104
						H (68)	0.023732

The total positive charge of the functionalized CuFe₂O₄ increases from 8.095008 to 9.1070457, while the total negative charge increases from − 7.09502 to −8.1070449 compared to the unmodified nanoparticles. This increase in both positive and negative charge magnitudes indicate a more polarized system upon modification, enhancing the electronic interactions between the nanoparticle surface and surrounding molecules, such as water and hydrate-forming gases. The enhanced polarization in the functionalized system contributes to improved gas adsorption and water structuring around the nanoparticles, promoting faster and more efficient hydrate nucleation and growth. These changes underscore the role of Bzc in the modification of the electronic environment of CuFe₂O₄, enhancing its suitability for applications in natural gas hydrate formation.

**Table 7:** Total Charge of NFCu and NFCu - Ch.

	NFCu	NFCu - Ch.
Total Positive Charge	8.095008	9.1070457
Total Negative Charge	−7.09502	−8.1070449
Total Net Charge	0.999988	1.0000008

**Fig. 14:**
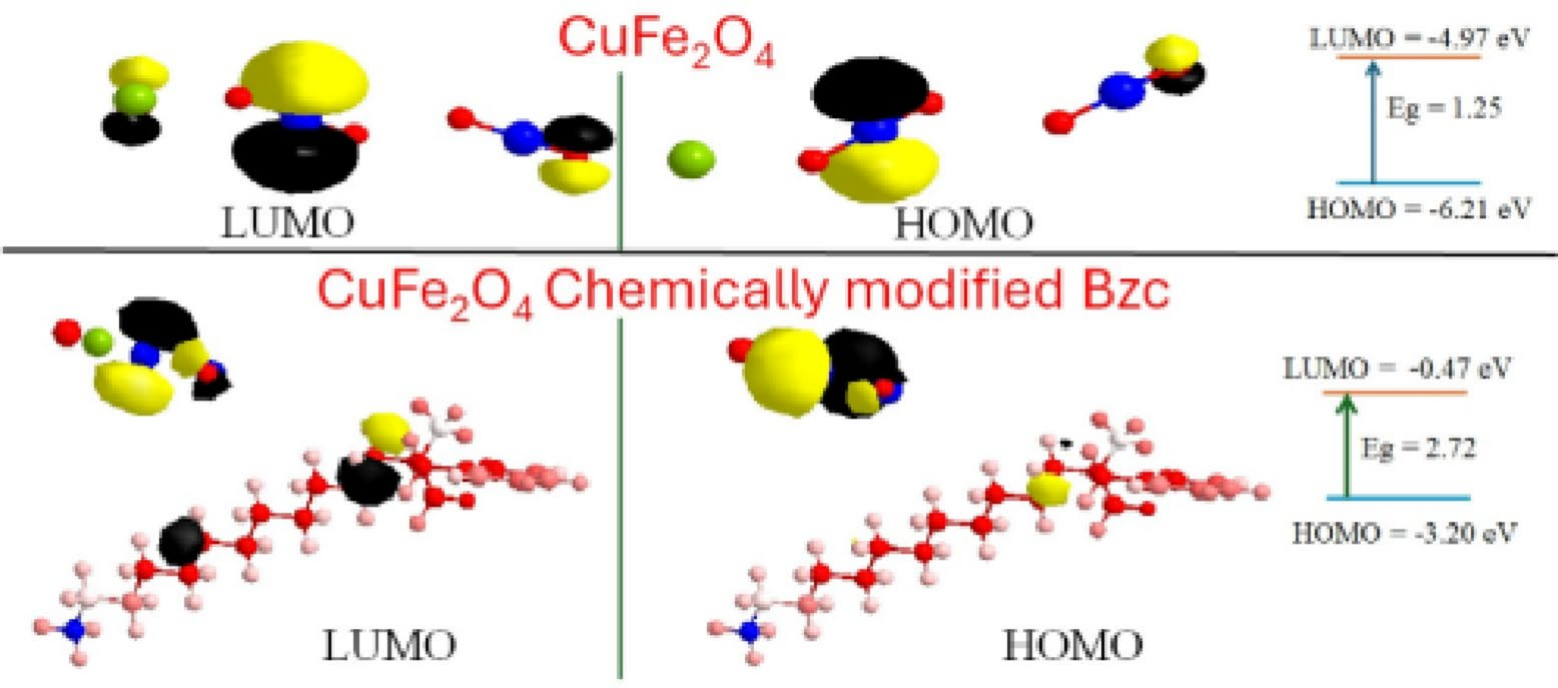
Electronic Distribution Charge (HOMO - LUMO) For NFCu and NFCu- Ch.

In Fig. 14 The charge distribution was carried out using Huckel theory. Copper ferrite (CuFe_2_O_4_) reveals a distinct polarity across the compound. The iron atoms (Fe(1) = −3.56074, Fe(4) = −3.53428) exhibit strong negative charges (blue regions), indicating the electron-rich sites. In contrast, the oxygen atoms (O(2) = 1.9601, O(3) = 1.71147, O(5) = 1.30773, O(6) = 2.38971) carry positive charges (red regions), reflecting electron-deficient sites. The copper atom (Cu(7) = 0.725998) also exhibits a slight positive charge (red region). This distribution suggests that CuFe_2_O_4_ has a polarized structure where negatively charged iron atoms are surrounded by positively charged oxygen and copper atoms. Such a charge arrangement facilitates electrostatic interactions, which play a role in surface reactivity and potential adsorption behavior. Upon the chemical adsorption of N-benzyl-N, N-dimethyldodecan-1-aminiumchloride (BAC) on CuFe_2_O_4_, the charge distribution changes significantly. While the iron atoms (Fe(1) = −3.56284, Fe(4) = −3.54059) retain their strong negative charges (blue regions), the oxygen atoms display a more varied distribution. O(2) = 0.430854 and O(3) **=** 0.263581 maintain positive charges (red regions) but at lower magnitudes, while O(5) **=** −0.476652 and O(6) **= -**0.390703 switch to negative charges (blue areas), suggesting a shift in electron density. The copper atom (Cu(7) = −0.0351799) also changes from a positive (red) to a slightly negative charge (blue). The organic component introduces new regions of positive and negative charges, most notably a strong positive charge on the nitrogen of ammonium groups (N(20) **=** 0.8797), which enhances local electrostatic interactions. Carbon atoms within the organic chain contribute to an overall increase in surface polarity. This redistribution implies a more complex electrostatic environment, which could affect the interaction of the compound with external molecules. The highest occupied molecular orbital (HOMO) and lowest unoccupied molecular orbital (LUMO) energy levels of pristine CuFe_2_O_4_ indicate moderate electronic stability and semiconducting behavior. The HOMO energy was calculated to be −6.21 eV, while the LUMO energy was −4.97 eV, yielding an energy gap of 1.25 eV. This relatively small band gap suggests that CuFe_2_O_4_ exhibits reasonable electronic conductivity and moderate chemical reactivity, enabling electron-transfer interactions with adsorbed species. Upon chemical modification with Bzc, significant changes in the electronic structure were observed. The HOMO energy shifted to −3.20 eV and the LUMO energy to −0.47 eV, resulting in an increased energy gap of 2.72 eV. This shift in frontier molecular orbital energies indicates enhanced electronic stability and modified charge distribution after surface functionalization. The change in energy levels reflects the strong electronic interaction between BAC and the CuFe_2_O_4_ surface, which alters the electron density and improves the material's electronic properties. These differences in charge distribution and frontier orbital energies directly influence water adsorption behavior. For pristine CuFe_2_O_4_, the smaller band gap (1.25 eV) facilitates moderate interaction with water molecules mainly through surface electrostatic attraction and weak coordination with metal sites. In contrast, the chemically modified CuFe_2_O_4_Bzc surface exhibits altered electronic density and a wider energy gap (2.72 eV), which enhances surface stability and promotes stronger electrostatic interactions with water molecules. The presence of positively charged ammonium groups in Bzc increases attraction with the oxygen atoms of water molecules, improving adsorption and retention. Additionally, the redistribution of electron density on the modified surface enhances the interaction between active metal centers and water molecules.

Overall, chemical modification of CuFe_2_O_4_ with Bzc significantly alters the frontier orbital energies and charge distribution, thereby improving electronic stability and enhancing adsorption of water molecules.

## Conclusion

This study explored the role of CuFe₂O₄ nanoparticles and their modified forms with benzalkonium chloride (Bzc) to improve natural gas hydrate (NGH) formation. The findings revealed that, the chemically modifying CuFe₂O₄ with Bzc significantly enhanced the hydrate formation kinetics by reducing induction time and increasing gas storage capacity compared to the unmodified form CuFe₂O₄ and the physically blend form. The Bzc-functionalized CuFe₂O₄ nanoparticles exhibited a remarkable reduction in the induction time to 5 min at 0.005 wt% NFCu-Ch., compared to 12 min for the unmodified CuFe₂O₄ at 0.02 wt%. Additionally, the gas storage capacity was significantly higher, reaching 0.35 moles of gas per mole of water at 0.005 wt%, representing a 191.67% increase over the unmodified nanoparticles. The hydrate formation rate was also notably enhanced due to the improving the surface characteristics which was provided by Bzc functional groups, which facilitated the nucleation and growth of hydrate formation. Furthermore, the modified nanoparticles improved NGH dissociation behavior, achieving up to 95% gas recovery compared to 81.51% for the unmodified CuFe₂O₄. These enhancements were attributed to the altered surface activity, increased electron transfer capabilities, and modified morphology of the nanoparticles, which promoted more effective gas adsorption and nucleation. Overall, the study highlights the potential of Bzc-modification for the CuFe₂O₄ nanoparticles as efficient promoters for the NGH formation, making them promising to use for the natural gas storage and transportation applications.

## Supplementary Information

Below is the link to the electronic supplementary material.

Supplementary Material 1

Supplementary Material 2

## Data Availability

The data supporting this study’s findings are available from the corresponding author upon reasonable request. Email: Eng_msalah1@yahoo.com.
