# Supplementary material for: Preparation of new modified CuFe₂O₄ nanoparticles by benzalkonium chloride as enhancer of natural gas hydrate formation
Source: Sci Rep. 2026 Apr 22;16:14634. doi: 10.1038/s41598-026-44211-2 (PMC13153427; doi:10.1038/s41598-026-44211-2)
Supplement: Supplementary file 1 — Supplementary Material 1 [file 41598_2026_44211_MOESM1_ESM.docx]

**Supplementary Material: Kinetic Analysis and Sample Calculations**

**Definition of Gas Consumption Rate**

The hydrate formation rate is defined as the instantaneous molar rate of gas molecules transitioning into the hydrate phase. As described in Equation (1) of the manuscript, this rate is determined through a step-by-step numerical method:

(1)

$\frac{\text{dn}}{\text{ }dt}$ = ( $\frac{\text{ d }\text{Δ}\text{n}\text{↓}\text{,}\text{H}\text{ }}{\text{dt }}$) t = $\frac{(\Delta n\downarrow,H \text{, t} + \Delta t \text{ — }\text{Δ}\text{n}\text{↓}\text{,}\text{H}\text{ t } )}{\Delta t\text{ }}$

Where:

- $\frac{\text{dn}}{\text{ }dt}$: Instantaneous gas consumption rate (mol/min).
- $\Delta n\downarrow,H \text{, t} + \Delta t$: Cumulative moles of gas consumed at time t (mol).
- $\text{Δ}\text{n}\text{↓}\text{,}\text{H}\text{ t}$ : moles of gas consumed at time t (mol)
- $\Delta t$: Time interval between measurements (5 min).

**Sample Calculations for dn/dt**

To demonstrate the numerical method, calculations for the first three-time intervals are provided below. These calculations use a water volume of 400 mL (n_w_ = 22.20 mol).

Example 1: Interval t_1_ (0 to 5 min)

- Cumulative gas at t=0: 0 mol
- Cumulative gas at t=5: 0.1585 mol

Calculation:

{dn}/{dt} = {0.1585 - 0}/ {5} =0.0317 mol/min

Example 2: Interval t_2_ (5 to 10 min)

- Cumulative gas at t=5: 0.1585$ mol
- Cumulative gas at t=10: 0.20805 mol

Calculation:

{dn}/{dt} = 0.20805 - 0.1585}/{5} = 0.00991 mol/min

Example 3: Interval t_3_ (10 to 15 min)

- Cumulative gas at t=10: 0.20805 mol
- Cumulative gas at t=15: 0.21255 mol

Calculation:

{dn}/{dt} = {0.21255 - 0.20805}/{5} = 0.0009 mol/min

**Detailed Kinetic Data (First 10 Points)**

The following table presents the calculated instantaneous rates and the normalized rates (per mole of water) for the first 45 minutes of the experiment.

| **Point** | **Time (min)** | **dn/dt (mol/min)** | **Normalized Rate (mol/mol$_w$/min)** |
| --- | --- | --- | --- |
| t_1_ | 0 to 5 | 0.0317 | 0.001428 |
| t_2_ | 5 to 10 | 0.00991 | 0.000446 |
| t_3_ | 10 to 15 | 0.0009 | 0.000041 |
| t_4_ | 15 to 20 | 0.0006 | 0.000027 |
| t_5_ | 20 to 25 | 0 | 0 |
| t_6_ | 25 to 30 | 0.00069 | 0.000031 |
| t_7_ | 30 to 35 | 0.0001 | 0.000005 |
| t_8_ | 35 to 40 | 0.00058 | 0.000026 |
| t_9_ | 40 to 45 | 0.0001 | 0.000005 |
| t_10_ | 45 to 50 | 0.00009 | 0.000004 |

*Note: Normalization is based on n_w_ = 22.2037$ mol of water.*

**Supplementary Material (Experimental Evidence)**

**Investigation of Nanoparticle Dispersibility and Stability**

To validate the stability of the nanofluids used in the hydrate formation experiments, the dependence of dispersibility on nanoparticle concentration was investigated using Zeta Potential and Electrical Conductivity measurements.

1. **Methodology**

The nanoparticles of unmodified CuFe_2_0_4_ and nanoparticles of chemically CuFe_2_0_4-_ Bzc were dispersed in deionized water at the experimental concentrations. Each sample underwent ultrasonication for 30 minutes to ensure initial uniformity.

- Zeta Potential: Measured using a Zetasizer to determine the electrokinetic potential at the colloidal sliding plane.
- Electrical Conductivity: Recorded to evaluate the ion mobility and potential agglomeration effects at higher weight percentages.

1. **Results and Discussion**

The experimental data reveals that chemically CuFe_2_0_4-_ Bzc at lower concentrations, the Zeta Potential remains highly negative, typically zeta ≈ 30 mV indicating good stability without aggregation. As the concentration increases over [0.005 wt% of chemically modified CuFe_2_0_4-_ Bzc], a decrease in the absolute Zeta Potential was observed, suggesting a reduction in electrostatic repulsion which may lead to incipient flocculation.


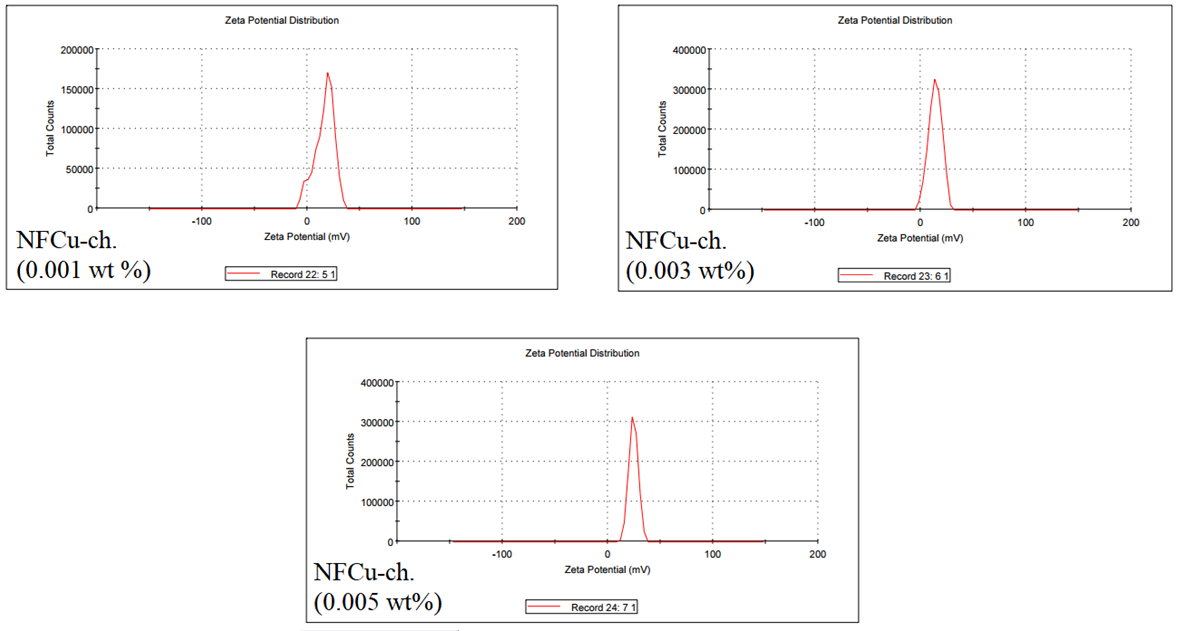


**Fig. 1. Zeta Potential Measurements for Different Concentration of Chemically CuFe_2_0_4-_ Bzc**
